# Cerebrospinal fluid findings in COVID-19: a multicenter study of 150 lumbar punctures in 127 patients

**DOI:** 10.1186/s12974-021-02339-0

**Published:** 2022-01-20

**Authors:** Sven Jarius, Florence Pache, Peter Körtvelyessy, Ilijas Jelčić, Mark Stettner, Diego Franciotta, Emanuela Keller, Bernhard Neumann, Marius Ringelstein, Makbule Senel, Axel Regeniter, Rea Kalantzis, Jan F. Willms, Achim Berthele, Markus Busch, Marco Capobianco, Amanda Eisele, Ina Reichen, Rick Dersch, Sebastian Rauer, Katharina Sandner, Ilya Ayzenberg, Catharina C. Gross, Harald Hegen, Michael Khalil, Ingo Kleiter, Thorsten Lenhard, Jürgen Haas, Orhan Aktas, Klemens Angstwurm, Christoph Kleinschnitz, Jan Lewerenz, Hayrettin Tumani, Friedemann Paul, Martin Stangel, Klemens Ruprecht, Brigitte Wildemann, in cooperation with the German Society for Cerebrospinal Fluid Diagnostics and Clinical Neurochemistry

**Affiliations:** 1 Molecular Neuroimmunology Group, Department of Neurology, University of Heidelberg, Heidelberg, Germany; 2 Department of Neurology, Charité—Universitätsmedizin Berlin, Corporate Member of Freie Universität Berlin and Humboldt-Universität zu Berlin, Berlin, Germany; 3 German Center for Neurodegenerative Diseases (DZNE) in Magdeburg, Magdeburg, Germany; 4 Neuroimmunology and Multiple Sclerosis Research Section, Department of Neurology, University Hospital Zurich, Zurich, Switzerland; 5 Department of Neurology and Center for Translational Neuro- and Behavioral Sciences (C-TNBS), University Medicine Essen, University of Duisburg-Essen, Essen, Germany; 6 IRCCS Ospedale Policlinico San Martino, Genoa, Italy; 7 Neurocritical Care Unit, Department of Neurosurgery and Institute of Intensive Care, University Hospital and University of Zurich, Zurich, Switzerland; 8 Department of Neurology, University of Regensburg, Regensburg, Germany; 9 Department of Neurology, DONAUISAR Klinikum Deggendorf, Deggendorf, Germany; 10 Department of Neurology, Medical Faculty, Heinrich-Heine-University Düsseldorf, Düsseldorf, Germany; 11 Department of Neurology, Center for Neurology and Neuropsychiatry, LVR-Klinikum, Heinrich-Heine-University Düsseldorf, Düsseldorf, Germany; 12 Department of Neurology, Ulm University, Ulm, Germany; 13 Medica Medical Laboratories Dr. F. Kaeppeli AG, Zurich, Switzerland; 14 Institute of Intensive Care Medicine, University Hospital and University of Zurich, Zurich, Switzerland; 15 Department of Neurology, School of Medicine, Technical University of Munich, Munich, Germany; 16 Department of Gastroenterology, Hepatology and Endocrinology, Hannover Medical School, Hannover, Germany; 17 Regional Referral Multiple Sclerosis Centre, Department of Neurology, University Hospital S. Luigi - Orbassano (I), Orbassano, Italy; 18 Department of Neurology, University Hospital Zurich, Zurich, Switzerland; 19 Clinic of Neurology and Neurophysiology, Medical Center University of Freiburg, Faculty of Medicine, University of Freiburg, Freiburg, Germany; 20 Department of Neurology, University Medical Center of the Johannes Gutenberg University Mainz, Mainz, Germany; 21 Department of Neurology, St. Josef-Hospital, Ruhr-University Bochum, Bochum, Germany; 22 Department of Neurology, Sechenov First Moscow State Medical University, Moscow, Russia; 23 Department of Neurology with Institute of Translational Neurology, University and University Hospital Münster, Münster, Germany; 24 Department of Neurology, Medical University of Innsbruck, Innsbruck, Austria; 25 Department of Neurology, Medical University of Graz, Graz, Austria; 26 Neuroinfectiology Group, Department of Neurology, University of Heidelberg, Heidelberg, Germany; 27 Specialty Hospital of Neurology Dietenbronn, Schwendi, Germany; 28 Experimental and Clinical Research Center, Max Delbrueck Center for Molecular Medicine and Charité—Universitätsmedizin Berlin, Berlin, Germany; 29 Clinical Neuroimmunology and Neurochemistry, Department of Neurology, Hannover Medical School, Hanover, Germany

**Keywords:** Coronavirus disease 2019 (COVID-19), Severe acute respiratory syndrome coronavirus type 2 (SARS-CoV-2), Neurological symptoms, Lumbar puncture, Cerebrospinal fluid (CSF), Oligoclonal bands, Blood-CSF barrier, Polymerase Chain reaction (PCR), SARS-CoV-2 antibodies, Antibody index, Autoantibodies, Cytokines, Central nervous system, Encephalopathy, Encephalitis, Guillain–Barré syndrome

## Abstract

**Background:**

Comprehensive data on the cerebrospinal fluid (CSF) profile in patients with COVID-19 and neurological involvement from large-scale multicenter studies are missing so far.

**Objective:**

To analyze systematically the CSF profile in COVID-19.

**Methods:**

Retrospective analysis of 150 lumbar punctures in 127 patients with PCR-proven COVID-19 and neurological symptoms seen at 17 European university centers

**Results:**

The most frequent pathological finding was blood-CSF barrier (BCB) dysfunction (median QAlb 11.4 [6.72–50.8]), which was present in 58/116 (50%) samples from patients without pre-/coexisting CNS diseases (group I). QAlb remained elevated > 14d (47.6%) and even > 30d (55.6%) after neurological onset. CSF total protein was elevated in 54/118 (45.8%) samples (median 65.35 mg/dl [45.3–240.4]) and strongly correlated with QAlb. The CSF white cell count (WCC) was increased in 14/128 (11%) samples (mostly lympho-monocytic; median 10 cells/µl, > 100 in only 4). An albuminocytological dissociation (ACD) was found in 43/115 (37.4%) samples. CSF l-lactate was increased in 26/109 (24%; median 3.04 mmol/l [2.2–4]). CSF-IgG was elevated in 50/100 (50%), but was of peripheral origin, since QIgG was normal in almost all cases, as were QIgA and QIgM. In 58/103 samples (56%) pattern 4 oligoclonal bands (OCB) compatible with systemic inflammation were present, while CSF-restricted OCB were found in only 2/103 (1.9%). SARS-CoV-2-CSF-PCR was negative in 76/76 samples. Routine CSF findings were normal in 35%. Cytokine levels were frequently elevated in the CSF (often associated with BCB dysfunction) and serum, partly remaining positive at high levels for weeks/months (939 tests). Of note, a positive SARS-CoV-2-IgG-antibody index (AI) was found in 2/19 (10.5%) patients which was associated with unusually high WCC in both of them and a strongly increased interleukin-6 (IL-6) index in one (not tested in the other). Anti-neuronal/anti-glial autoantibodies were mostly absent in the CSF and serum (1509 tests). In samples from patients with pre-/coexisting CNS disorders (group II [*N* = 19]; including multiple sclerosis, JC-virus-associated immune reconstitution inflammatory syndrome, HSV/VZV encephalitis/meningitis, CNS lymphoma, anti-Yo syndrome, subarachnoid hemorrhage), CSF findings were mostly representative of the respective disease.

**Conclusions:**

The CSF profile in COVID-19 with neurological symptoms is mainly characterized by BCB disruption in the absence of intrathecal inflammation, compatible with cerebrospinal endotheliopathy. Persistent BCB dysfunction and elevated cytokine levels may contribute to both acute symptoms and ‘long COVID’. Direct infection of the CNS with SARS-CoV-2, if occurring at all, seems to be rare. Broad differential diagnostic considerations are recommended to avoid misinterpretation of treatable coexisting neurological disorders as complications of COVID-19.

**Supplementary Information:**

The online version contains supplementary material available at 10.1186/s12974-021-02339-0.

## Background

COVID-19, first described in December 2019, is an infectious disease caused by severe acute respiratory syndrome coronavirus type 2 (SARS-CoV-2). Acute COVID-19 has been reported to affect—directly or indirectly—the nervous system in a substantial number of cases [1–6]. A broad spectrum of neurological manifestations, ranging from mild hyposmia and dysgeusia to life-threatening conditions such as acute encephalopathy and stroke, have been described in association with COVID-19 [1–3].

Cerebrospinal fluid (CSF) analysis is a diagnostic mainstay in neurology. However, limited information on CSF findings in patients with COVID-19 is currently available. Many of the studies published so far are restricted in terms of the number of patients included [7, 8] and/or the parameters assessed [9, 10], are based on a review of the literature [11], or report experience from single centers [12]. Comprehensive data from large-scale multicenter studies that take into account a wide spectrum of parameters, including CSF white cell counts (WCC) and cytology, quantitative and qualitative evidence of intrathecal IgG, IgM and IgA synthesis (including Reiber diagrams [‘reibergrams’]), markers of blood-CSF barrier (BCB) dysfunction, total protein, l-lactate, glucose, and SARS-CoV-2 CSF polymerase chain reaction (PCR), antibody indices (AI), autoantibody findings, and cytokine levels, are widely missing so far. On behalf of the German Society for CSF Diagnostics and Clinical Neurochemistry  (DGLN) we conducted a systematic analysis of CSF findings from 150 lumbar punctures in 127 patients with PCR-proven COVID-19 and neurological symptoms. Patients were stratified according to the type and severity of the neurological symptoms, acuity, co-/preexisting neurological conditions, and treatment status.

## Patients and methods

### Patients

The results of 150 lumbar punctures (LPs) in 127 adult patients with COVID-19 and neurological symptoms were obtained from the patient records and analyzed retrospectively. LPs were performed between 03/2020 and 01/2021. In all cases, SARS-CoV-2 infection was confirmed by PCR (nasopharyngeal/nasal swab/sputum/tracheal secretion). All patients were diagnosed with COVID-19 at 17 German (Heidelberg, Berlin, Essen, Bochum, Düsseldorf, Freiburg, Hanover, Mainz, Münster, Munich, Regensburg, Ulm), Austrian (Graz, Innsbruck), Italian (Pavia, Orbassano) and Swiss (Zurich) university hospitals. See Additional file 1: table s1 for a summary of the patients' demographic and clinical features.

Eleven patients (19 CSF samples) were excluded from primary analysis due to pre-/coexisting inflammatory (multiple sclerosis [MS] in 3, acute herpes simplex virus [HSV] type 1 encephalitis [HSVE] in 1, acute varicella zoster virus [VZV] encephalitis in 1, anti-Yo-associated autoimmune encephalitis in 1, chronic viral meningitis of unknown etiology in 1), neoplastic (primary CNS lymphoma in 1, meningeal carcinomatosis in 1), or cerebrovascular (severe subarachnoid hemorrhage [SAH] resulting in severe blood contamination of the CSF sample in 2) CNS conditions known to cause CSF alterations and considered by the treating physicians to be most likely not related to COVID-19; these patients were analyzed separately as ‘group II’ (f:m ratio = 1:0.8; median age 54 years, range 29–76).

Of the remaining patients (N = 116; ‘group I’), 90 were male (f:m ratio = 1:3.5). Eighty-eight percent were of Caucasian, 5% of African, 4% of Turkish or Middle-Eastern, and 3% of Asian origin. Overall, 131 LPs were performed in these 116 patients following onset of COVID-19 (one sample available from 102 patients, two samples available from 13, and three samples from 1). The median age at LP was 65 years (range 19–89). The median time between first SARS-CoV-2-PCR-positive swab and first LP was 12 days (percentile range [0.1–0.9] 0–44), and the median time between LP and onset of the first neurological symptoms that prompted it was 5 days (percentile range 1–26). The median time between the first SARS-CoV-2-PCR-positive swab and the onset of neurological symptoms was 7 days. Neurological manifestations at the time of LP included encephalopathy, disturbed consciousness, or delayed wake-up reaction (63 LPs), seizures or epilepsy-like EEG changes (27 LPs), cerebral ischemia or bleeding (14 LPs), myelitis (3 LPs), other CNS manifestations (25 LPs; including cerebellar ataxia, sensorimotor symptoms of unknown cause, and cognitive impairment), peripheral neuropathy (22 LPs; including Guillain-Barré syndrome (GBS) [at least 6 LPs]), cranial nerve symptoms (at least 10 LPs; including anosmia and dysgeusia), and headache (at least 13 LPs; including 3 × isolated severe headache and 1 × headache and nausea) (multiple manifestations per patient possible). The presence of neurological disease was supported by paraclinical evidence from magnetic resonance imaging (MRI) or computed tomography (CT) at the time of LP in 94 cases, by EEG in 35, and by ENG/EMG in 18. At the time of LP, most patients had symptoms attributable to brain or spinal cord involvement (N = 108 [82%] samples; ‘brain/spinal cord [B/SC] subgroup’), while some had exclusively hyposmia, dysgeusia, isolated cranial nerve involvement, peripheral nerve damage, and/or headache (N = 22 samples [17%]; ‘peripheral nerve/cranial nerve/headache [PN/CN/H] subgroup’) (no exact classification possible in 1 case). The neurological symptoms present at the time of LP were coarsely classified by the treating neurologists as ‘severe’ for 57.9% of LPs, ‘moderate’ for 24.8%, and ‘mild’ for 17.4%, and samples were stratified accordingly for further analysis; at least 57% of the samples were taken during or within 1 week before or after ICU treatment and 57% during or within 1 week before or after ventilation. Serum IgG antibodies against SARS-CoV-2 were determined by enzyme-linked immunosorbent assay (ELISA) in 58 samples from 56 patients in group I (as part of routine clinical workup) and were positive in 51 (88%) samples from 49 (87.5%) patients (median 28 days since non-neurological disease onset; percentile range [0.1–0.9] 12–45) and negative in 7 samples from 6 patients (median only 5 days, up to 12 days; *N* = 7); in the remaining patients, either serum antibody testing was not done because of the short time interval between COVID-19 onset and LP or data were not available retrospectively.

For the purpose of this study, samples obtained within 14 days of the onset of a patient’s neurological symptoms were classified as ‘acute’ (75% of all samples in the ‘B/SC subgroup’ and 89% in the ‘PN/CN/H subgroup’). To avoid bias due to differences in acuity, some subgroup analyses were restricted to ‘acute’ samples (‘acute B/SC subgroup’, ‘acute PN/CN/H subgroup’). The study was approved by the review boards of the participating centers and patients or their legal representatives gave written informed consent. LPs were performed for diagnostic purposes in all cases; no samples were obtained for use in this study.

Due to the retrospective nature of this study, not all data of interest were available for all patients and samples. Accordingly, absolute patient numbers and/or sample numbers differed among subanalyses (e.g., CSF white cell counts were determined in more patients and samples than anti-neural autoantibodies). No data were excluded from analysis unless explicitly stated (e.g., for not meeting subgroup criteria).

### Methods

Methods were adopted from our previous studies on CSF findings in inflammatory CNS disorders [13–18] adhering to the German Guidelines on CSF diagnostics of the German Society for CSF Diagnostics and Clinical Neurochemistry and the German Society of Neurology [19–21].

#### Evaluation of humoral immune response

Oligoclonal IgG bands (OCB) were assessed by isoelectric focusing and evaluated according to an international consensus [22]. Immunoglobulins and albumin were measured immunonephelometrically. Quantitative expressions of the intrathecal humoral immune response were based on calculation of the CSF/serum quotients QIgG, QIgM, and QIgA with QIg = Ig_CSF_[mg/l]/Ig_serum_[g/l]. The upper limits of the respective reference ranges, Q_lim_(IgG), Q_lim_(IgM), and Q_lim_(IgA), were calculated against QAlb according to Reiber’s revised hyperbolic function [23]. Values for QIg exceeding Q_lim_(Ig) were considered to indicate intrathecal immunoglobulin synthesis [23]. The fraction (in %) of intrathecally produced Ig (Ig-IF) and the absolute amount of locally, i.e., intrathecally, produced immunoglobulins (Ig-loc) were calculated according to the following formulas: Ig-IF[%] = [QIg−Q_lim_(Ig)] × Ig_serum_ × 100 and Ig-loc[mg/L] = [QIg−Q_lim_(Ig)] × Ig_serum_, respectively [23].

#### Antibody indices

The intrathecal synthesis of antibodies was detected by calculation of the corresponding anti-microbial AI: AI = QIg[spec]/QIg[total], if QIg[total] < Q_lim_(Ig), and AI = QIg[spec]/Q_lim_(Ig), if QIg[total] > Q_lim_(Ig) with QIg[spec] = IgG[spec]_CSF_/IgG[spec]_serum_ and QIgG[total] = IgG[total]_CSF_/IgG[total]_serum_)[24]. The upper reference range of QIg, Q_lim_(Ig), was calculated according to Reiber’s formulas [23]. AI values > 1.5 were considered positive [24].

#### Evaluation of blood–CSF barrier function

The CSF/serum albumin quotient, QAlb = Alb_CSF_[mg/l]/Alb_serum_[g/l], was used to assess BCB function. As the upper reference limit of QAlb is age dependent, Q_lim_(Alb) was calculated as (4 + ( *a*/15)) × 10^–3^ with *a* representing patient´s age according to Reiber et al. [25]. Dysfunction of the BCB was defined as QAlb > Q_lim_(Alb) [24].

#### Cytological examination, CSF total protein, and CSF l-lactate

A CSF WCC > 5/µl was classified as ‘increased’ [20]. An age-dependent reference range for CSF l-lactate was employed (16–50: 2.1 mmol/l, > 50: 2.6 mmol/l) [20]. The upper reference limit for CSF total protein was set at 0.45 mg/ l [20].

#### Other markers

Reference ranges provided by the performing laboratories were used to assess the frequency of samples with elevated CSF and/or serum concentrations or quotients, respectively, for CSF interleukin (IL) 6, serum IL-6, serum IL-8, serum IL-1beta, serum IL-10, serum IL-1RA, serum soluble IL-2 receptor (sIL2R), serum tumor necrosis factor alpha (TNF-alpha), and interferon gamma (IFN-gamma), CSF and serum free kappa and lambda light chains, and CSF and serum SARS-CoV-2-IgG. For CSF markers with no well-defined normal ranges (IL-8, TNF-alpha, and IFN-gamma), median and ranges were given and descriptively compared with CSF concentrations reported in the previous literature in healthy controls or patients with non-inflammatory neurological diseases [26–34].

#### Statistics

Samples were analyzed in total as well as after stratification according to disease status and treatment status. Fisher’s exact test, Mann–Whitney *U* test and Kruskal–Wallis test were used to assess the statistical significance of differences between groups. Spearman’s rho was used to assess correlations. Due to the exploratory nature of this study, no correction for multiple testing was applied other than Dunn’s post-test. *P* values < 0.05 were considered statistically significant. Reiber diagrams (‘reibergrams’) [21, 24] were generated using *CSF Research Tool* v3.0 (CoMed GmbH, Soest, Germany) and *FLC-K Statistics* v1.02 (Albaum IT Solutions, Möhnesee, Germany).

## Results

### CSF findings in group I

#### Blood–CSF barrier function

**Fig. 1:**
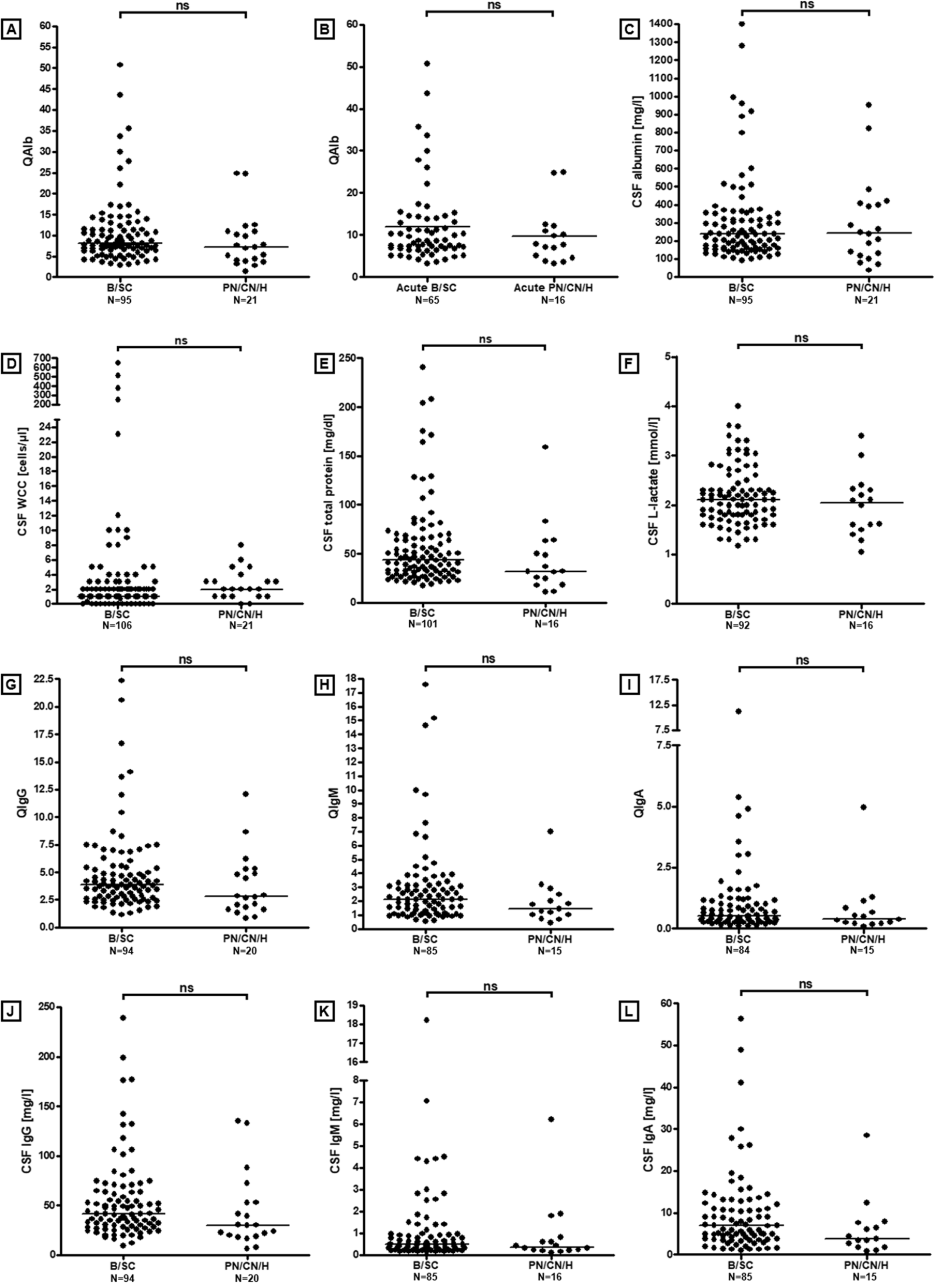
Albumin CSF/serum ratios (**A**,** B**) and CSF concentrations (**C**), CSF white cell counts (**D**), CSF total protein (**E**) and CSF L-lactate (**F**) concentrations and IgG, IgM and IgA CSF/serum ratios (**G**-**I**) and CSF concentrations (**J**-**L**) in patients with COVID-19 and neurological symptoms. Although some parameters were more markedly or more frequently altered in the ‘B/SC subgroup’ than in patients from the ‘PN/CN/H subgroup’, the differences were not statistically significant. N indicates the number of samples tested. Note that the figure shows all samples with available data; data stratified according to disease duration at the time of LP can be found in Tables 1, 2, 3, 4, 5. Solid lines indicate medians. *B/SC* brain spinal cord, *PN/CN/H* peripheral nerve/cranial nerve/headache only; *IgG/A/M* immunoglobulin G/A/M, *QIgG/A/M* CSF/serum IgG/A/M ratios, *QAlb* CSF/serum albumin ratio

An elevated CSF/serum ratio for albumin (QAlb), indicating dysfunction of the BCB, was found in 58/116 (50%) samples with available data in group I and was present at least once in 54/106 (50.9%) patients tested for this marker in group I. In those patients with BCB dysfunction, QAlb ranged from 6.72–50.8 × 10^–3^ (median 11.4) (Table 1 and Fig. 1A, B).

**Table 1:** Blood–CSF barrier function, CSF and serum albumin, CSF total protein, and CSF L-lactate in group I

	Units	Total	≤ 14 d	> 14 d	Acute B/SC subgroup	Acute PN/CN/H subgroup
Blood-CSF barrier function						
QAlb > QAlb(lim)	Samples	58/116 (50%)	46/82 (56.1%)	10/21 (47.6%)	37/65 (56.9%)	8/16 (50%)
QAlb, all LPs	–	7.9 (1.5–50.8;116)	8.6 (3.2–50.8;82)	8.1 (3–14.3;21)	8.6 (3.3–50.8;65)	7.7 (3.2–24.8;16)
QAlb, if positive	–	11.4 (6.72–50.8;58)	11.8 (6.72–50.8;46)	11.1 (8.09–14.3;10)	12.9 (6.7–50.8;37)	11.5 (6.9–24.8;8)
Alb CSF	mg/l	238 (38–1400;116)	243 (80–1400;82)	206 (102–516;21)	240 (106–1400;65)	276 (80–951;16)
Alb serum	g/l	28.9 (14–54.8;117)	30.25 (15.4–54.8;82)	31.9 (14–51.5;21)	28.3 (15.4–48.4;65)	36.4 (19.3–54.8;16)
Albuminocytological dissociation	Samples	43/115 (37.4%)	32/78 (41%)	7/23 (30.4%)	37/99 (37.4%)	6/16 (37.5%)
Combined intrathecal synthesis and BCB disruption	Samples	0/57 (0%)	0/45 (0%)	0/10 (0%)	0/36 (0%)	0/8 (0%)
CSF total protein						
CSF TP, elevated	Samples	54/118 (45.8%)	41/78 (52.6%)	8/23 (34.8%)	35/66 (53%)	6/11 (54.5%)
CSF TP, all LPs	mg/dl	41.75 (11–240.4;118)	46.55 (17.5–240.4;78)	34 (18.7–75.1;23)	46.55 (17.5–240.4;66)	48.6 (17.7–158.8;11)
CSF TP, if elevated	mg/dl	65.35 (45.3–240.4;54)	65.7 (45.3–240.4;41)	57.6 (45.9–75.1;8)	70 (45.3–240.4;35)	63.65 (48.6–158.8;6)
CSF TP, > 100 mg/dl	Samples	12/118 (10.2%)	10/78 (12.8%)	0/23 (0%)	9/66 (13.6%)	1/11 (9.1%)
CSF L-lactate						
CSF L-lactate, elevated	Samples	26/109 (23.9%)	20/74 (27%)	3/21 (14.3%)	17/62 (27.4%)	2/11 (18.2%)
CSF L-lactate, all LPs	mmol/l	2.1 (1.05–4;109)	2.1 (1.28–4;74)	2 (1.05–3.04;21)	2.1 (1.3–4;62)	2 (1.28–3.4;11)
CSF L-lactate, if elevated	mmol/l	3.04 (2.2–4;26)	3.02 (2.2–4;20)	3.03 (2.79–3.04;3)	2.25 (1.3–4;33)	2.2 (1.4–3.4;5)
CSF L-lactate, > 3 mmol/l	Samples	14/109 (12.8%)	10/74 (13.5%)	2/21 (9.5%)	8/62 (12.9%)	1/11 (9.1%)

Results are given as medians (with ranges and sample or patient numbers in brackets) and frequencies (with percentages in brackets), respectively. Note that columns 6 and 7 in Tables 1, 2, 3, 4, 5 refer to samples obtained within 14 days after neurological onset (‘acute B/SC subgroup’ and ‘acute PN/CN/H subgroup’, as defined in the *Patients* section); non-stratified data on the total ‘B/SC subgroup’ and the total ‘PN/CN/H subgroup’ can be found in the *Results* section and Fig. 1. *Alb* albumin, *BCB* bloodCSF barrier, *B/SC* brain/spinal cord, *LP* lumbar puncture, *PN/CN/H* peripheral nerve/cranial nerve/headache only, *QAlb* CSF/serum albumin ratio, *TP* total protein.

The frequency of BCB dysfunction was slightly higher in the ‘acute B/SC subgroup’ (56.9% [37/65]) than in the ‘acute PN/CN/H subgroup’ (50% [8/16]), as was median QAlb (8.6 [*N* = 65] vs. 7.7 [*N* = 16] if all samples with available data were considered, and 13 [*N* = 36] vs. 10.9 [*N* = 9] if only those with elevated QAlb were considered), but the differences did not reach statistical significance (Table 1 and Fig. 1A, B).

**Fig. 2:**
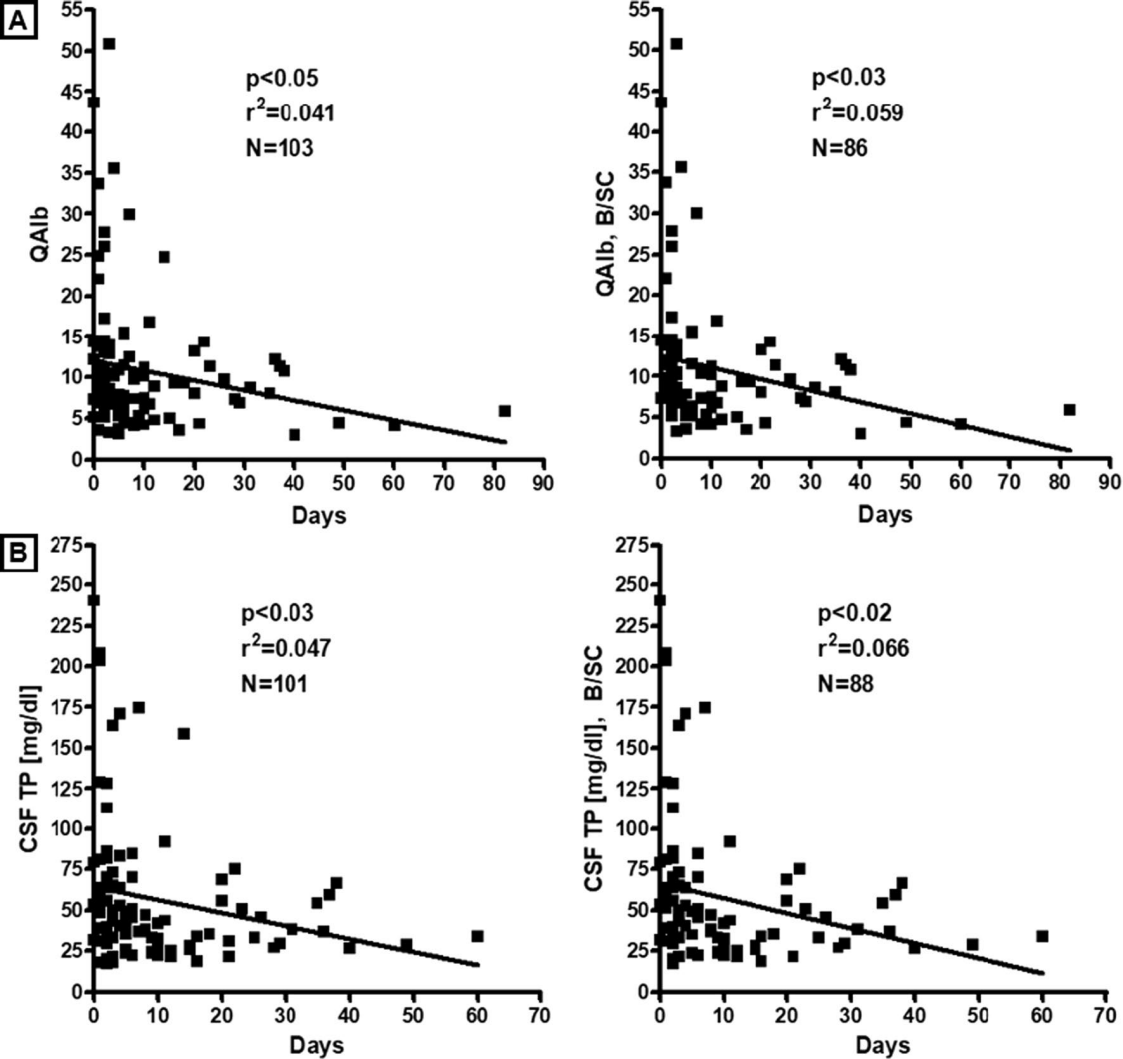
Correlation analyses for QAlb (**A**) and CSF total protein (**B**), respectively, and days since onset of the neurological symptoms in the total cohort and in the B/SC subgroup. Although a mildly significant correlation was found for both parameters, it should be noted that both parameters were still pathologically altered > 14 and even > 30 days after onset of the neurological symptoms in a subset of cases (see Results section and Table 1 for details). *B/SC* brain/spinal cord, *N* number of samples; *QAlb* albumin CSF/serum ratio, *TP* total protein

Although QAlb values decreased over time, both in the total cohort (*r* = − 0.201, *p* < 0.05; *r*^2^ = 0.041, *p* < 0.05; *N* = 103 samples with available data, from 95 patients) and in the B/SC subgroup (*r* = − 0.278, *p* < 0.03; *r*^2^ = 0.059, *p* < 0.03; *N* = 86 samples with available data, from 78 patients) (Fig. 2A), BCB dysfunction was still frequently present in samples taken > 14 days after onset of the neurological symptoms (56.1% [46/82 samples from 79 patients] ≤ 14 d, vs. 47.6% [10/21 samples from 21 patients] > 14 d; Table 1), including in 5/9 (55.6%) samples from 9 patients in whom neurological symptoms had started > 30 days before the respective LP (delirium, disturbed consciousness or delay in recovery of consciousness after ventilation in 4, with signs of epileptic activity in 2 and evidence of brain infarction in 1; ataxia, paresis, dysesthesia and urinary retention of unknown cause in 1).

Median serum albumin concentrations did not differ significantly between samples from patients with or without elevated QAlb values (28.35 mg/dl [range 15.4–47.9; N = 58] vs. 31.65 mg/dl [range 14–54.8; *N* = 58]), arguing against ICU-associated hypalbuminemia as a cause of QAlb elevation.

### CSF total protein

**Fig. 3:**
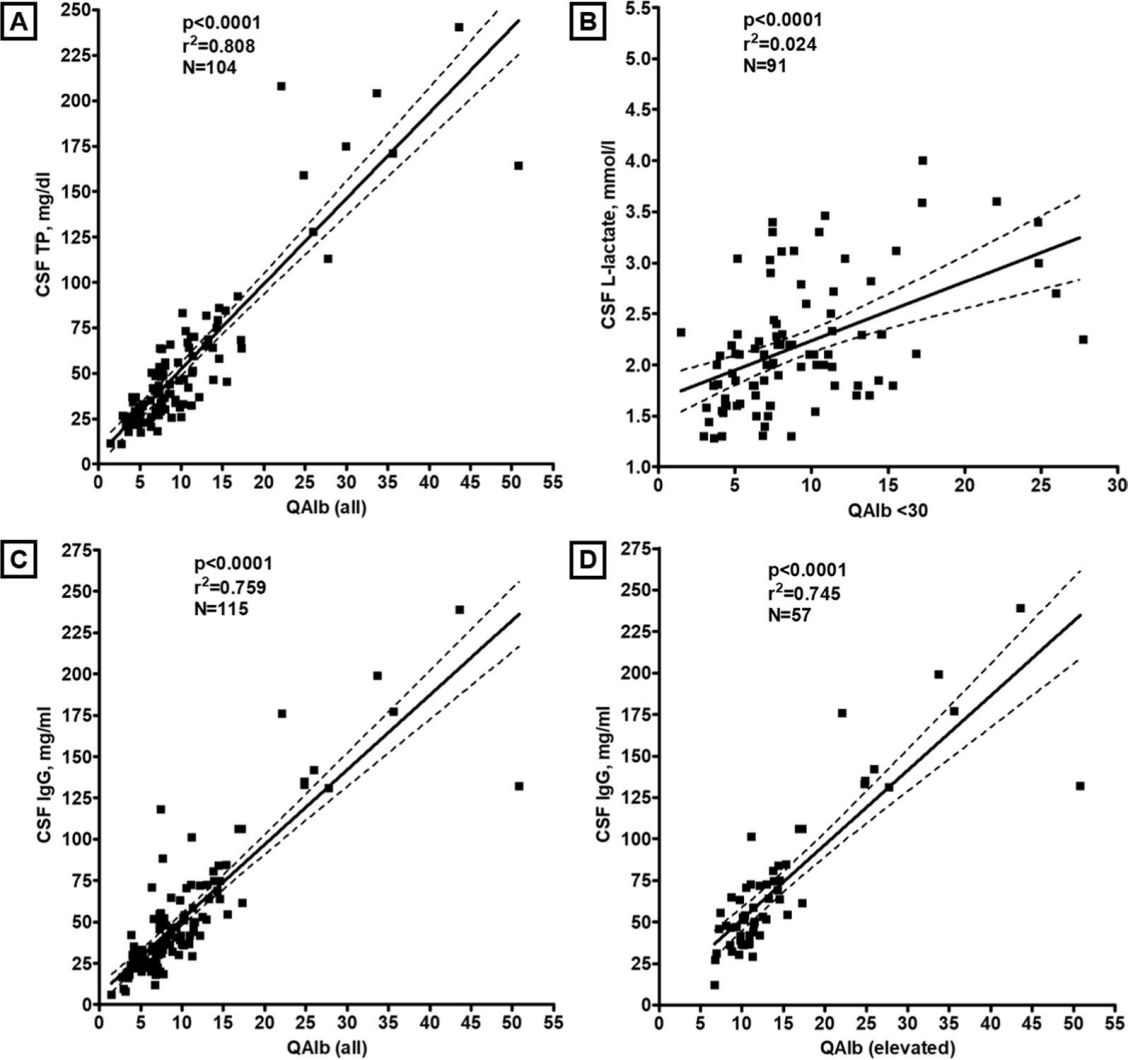
Regression analyses of CSF total protein (**A**), CSF L-lactate (after exclusion of samples with very high QAlb) (**B**) and CSF IgG concentrations (**C**-**D**), respectively, and QAlb, demonstrating a close relationship between these parameters and QAlb. Solid lines indicate medians. Dotted lines represent the upper and lower 95% confidence bands of the regression line. *IgG* immunoglobulin G, *N* number of samples, *QAlb* albumin CSF/serum ratio, *TP* total protein

TP concentrations in the CSF were elevated in 54/118 (45.8%) samples tested in group I (median 65.35 mg/dl; range 45.3–240.4) (Fig. 1E). As expected, TP levels were closely related to QAlb as detected by regression analysis (*r* = 0.899, *p* < 0.00001; *r*^2^ = 0.808, *p* < 0.00001; *N* = 104 samples with available data, from 94 patients) (Table 1 and Fig. 3A). In total, CSF TP levels were elevated at least once in 48/104 (46.2%) patients tested. Elevated CSF TP levels were > 45 and < 50 mg/dl (“borderline”) in 8/54 (14.8%) samples, ≥ 50 and ≤ 100 mg/dl in 34/54 (63%), > 100 and ≤ 150 mg/dl in 5/54 (9.3%), and exceeded 150 mg/dl in 7/54 (13%). However, CSF TP levels were elevated not only during the first 14 days after onset of the neurological symptoms (41/78 [52.6%] samples) but also later (8/23 [34.8%] samples) (Table 1), which is in line with the fact that QAlb remained elevated > 14 days in several cases as well. The frequency of CSF TP elevation did not differ between the acute ‘PN/CN/H subgroup’ and the acute ‘B/SC subgroup’, and median CSF TP levels did not differ between the two subgroups either; Table 1). As QAlb, CSF TP levels were negatively correlated with the time since onset of the neurological symptoms both in the total cohort (*r* = − 0.216, *p* < 0.03; *r*^2^ = 0.047, *p* < 0.03; *N* = 101 samples with available data, from 90 patients) and in the ‘B/SC subgroup’ (*r* = − 0.257, *p* < 0.02; *r*^2^ = 0.066, *p* < 0.02; *N* = 88 samples with available data, from 77 patients) (Fig. 2B).

### Cellular immune response

Only 14/128 (10.9%) samples from 12 different patients exhibited an increased CSF WCC, with a median of 10 cells/µl (Table 2 and Fig. 1D) (WCC not determined in 3 samples). Only 6 LPs from 5 patients yielded WCC counts > 10 cells/µl. In three of the 14 samples with pleocytosis, blood contamination was noted (1500, 5000, and 15,500 erythrocytes/µl, respectively). Although WCC are routinely corrected for blood contamination, the rules applied (e.g., reduction by 1 leukocyte/1000 erythrocytes) are rather crude, which renders it possible that the WCC were nevertheless falsely elevated in these few cases, all the more as the reported WCCs were low. If these samples are excluded, pleocytosis was present in only 11/128 (8.6%) samples with available data. The median WCC was higher in the ‘B/SC subgroup’ than in the ‘PN/CN/H subgroup’, but the difference did not reach statistical significance (Table 2 and Fig. 1D).

**Table 2:** CSF white cell counts and cytology results in group I. WCC in the various subgroups

	Units	Total	≤ 14 d	> 14 d	Acute B/SC subgroup	Acute PN/CN/H subgroup
Pleocytosis	Samples	14/128 (10.9%)	12/88 (13.6%)	2/26 (7.7%)	11/71 (15.5%)	1/16 (6.3%)
WCC, all samples	Cells/µl	2 (0–651;128)	2 (0–651;88)	1 (0–8;26)	2 (0–651;70)	2 (0–8;16)
WCC, if elevated	Cells/µl	10 (6–651;14)	11 (8–651;12)	7 (6–8;2)	12 (8–651;11)	8 (8–8;1)
WCC, ≥ 100	Samples	4/128 (3.1%)	4/88 (4.5%)	0/26 (0%)	4/71 (5.6%)	0/16 (0%)
WCC, if ≥100	Cells/µl	441.5 (247–651;4)	441.5 (247–651;4)	n.a. (n.a.;0)	441.5 (247–651;4)	n.a. (n.a.;0)
Lymphocytes	Samples	66/80 (82.5%)	47/56 (83.9%)	14/18 (77.8%)	36/44 (81.8%)	10/11 (90.9%)
Monocytes	Samples	67/80 (83.8%)	49/56 (87.5%)	13/18 (72.2%)	37/44 (84.1%)	11/11 (100%)
Neutrophils	Samples	17/80 (21.3%)	14/56 (25%)	2/18 (11.1%)	10/44 (22.7%)	4/11 (36.4%)
Eosinophils	Samples	0/80 (0%)	0/56 (0%)	0/18 (0%)	0/44 (0%)	0/11 (0%)
Basophils	Samples	0/80 (0%)	0/56 (0%)	0/18 (0%)	0/44 (0%)	0/11 (0%)
Plasma cells	Samples	2/80 (2.5%)	1/56 (1.8%)	1/18 (5.6%)	1/44 (2.3%)	0/11 (0%)
Lymphoid cells	Samples	7/80 (8.8%)	5/56 (8.9%)	2/18 (11.1%)	3/44 (6.8%)	2/11 (18.2%)
Macrophages	Samples	2/80 (2.5%)	2/56 (3.6%)	0/18 (0%)	1/44 (2.3%)	1/11 (9.1%)
No pleocytosis	Samples	114/128 (89.1%)	76/88 (86.4%)	24/26 (92.3%)	60/71 (84.5%)	15/16 (93.8%)

Results are given as medians (with ranges and sample numbers in brackets) and frequencies (with percentages in brackets), respectively

*B/SC* brain/spinal cord, *CSF* cerebropinal fluid, *PN/CN/H* peripheral nerve/cranial nerve/headache only, *WCC* white cell count

Lymphocytes were the predominant cell type in patients with pleocytosis and were present in 10/10 samples with available cytological data (accounting for up to 99% of all cells), monocytes in 9/10, activated lymphocytes in 3/10, neutrophils in 3/10 and plasma cells in 1/10; eosinophils and basophils were absent in all. Blood contamination was excluded in 9/10 (no data available in 1), including in the few with neutrophil pleocytosis.

The WCC exceeded 100 cells/µl in only 4 samples from 3 patients. The diagnoses in these cases included “fever, acute aphasia, apathy” (651 cells/µl at first LP and 373 cells/µl 7 days later; lymphomonocytic; CSF l-lactate 2.8 and 2.6 mmol/l; glucose CSF/serum ratio 63%; massive BCB dysfunction; LP performed 5 and 12 days after onset of non-neurological symptoms and 1 and 8 days after onset of neurological symptoms; almost complete recovery); “meningitis, fever, and moderate headache” (510 cells/µl; 68% neutrophils; CSF l-lactate 3.3 mmol/l; glucose CSF serum ratio 53%; BCB dysfunction; LP performed around 26 days after onset of non-neurological and 5 days after onset of neurological symptoms; full recovery); and “headache, meningism, cognitive impairment, later motor aphasia, flexion synergy” (9 cells/µl 3 days and 247 cells/µl 5 days after onset of the neurological symptoms; 85 and 92% lymphocytes, respectively, 12 and 4% neutrophils, 2 and 3% monocytes, and 3% plasma cells and 1% activated lymphocytes at second LP; full recovery).

Diagnoses in the remaining patients with pleocytosis, all with only slightly elevated WCC, included encephalopathy with seizures in two (8 and 23 cells/µl, respectively), multifocal myelitis with paraplegia/paraparesis in two (10 and 8 cells/µl, respectively), multiple brain infarctions in one (10 cells/µl), GBS (6 cells/µl), paresis, ataxia and dysesthesia in one (8 cells/µl), and abducens palsy in one (8 cells/µl).

While WCC > 100 occurred only in the ‘B/SC subgroup’, median WCCs did not differ between the acute ‘B/SC subgroup’ (2 cells/µl) and the acute ‘PN/CN/H subgroup’ (2 cells/µl), reflecting the low overall frequency of pleocytosis in both subgroups. The pleocytosis rate was slightly higher in the former group but the difference did not reach statistical significance.

### Albuminocytological dissociation

A so-called “albuminocytological dissociation” (ACD), i.e., elevated CSF TP in the absence of CSF pleocytosis, was found in 43/115 (37.4%) samples or 41/101 patients with available data (median TP concentration 64 mg/dl, up to 164.1 mg/dl). This included not only 6 samples from the ‘PN/CN/H’ subgroup but also 37 samples from the ‘B/SC subgroup’. The number increased only slightly (*N* = 45) when allowing for mild pleocytosis (≤ 10 cells/µl). Of all samples with elevated CSF TP and available WCC (N=53), 81.3% exhibited an ACD.

If ACD is defined with reference to albumin instead of TP, 47 samples with elevated QAlb (from 47 different patients) of 58 tested (81%) showed an ACD (including 9/10 samples tested from the ‘PN/CN/H subgroup’ and 38/48 (79.2%) samples from the ‘B/SC subgroup’). ACD was associated with stroke in only five of these patients and in 2 with (micro)hemorrhages. If not only samples with BCB dysfunction but all samples with available data are considered, ACD was present in 47/113 (41.6%) samples.

### CSF l-lactate

Of note, CSF l-lactate levels were increased in 26/109 (23.9%) CSF samples tested (and at least once in 25/97 [25.8%] patients tested), with a median concentration of 3.04 mmol/l (range 2.2–4) (Fig. 1F). Elevated l-lactate levels were found more frequently in samples taken during the first 14 days after onset of neurological symptoms than in samples taken later (27%, *N* = 74 vs. 14.3%, *N* = 21); however, the difference did not reach statistical significance (Table 1).

Elevation of CSF l-lactate levels was observed at slightly higher frequency in the acute ‘B/SC subgroup’ than in the acute ‘PN/CN/H subgroup’ (27.4%, *N* = 62 vs. 18.2%, *N* = 11; *p* = n.s.). Median CSF l-lactate concentrations did not differ between the two subgroups (2.1 vs. 2 mmol/l) (Table 1).

Of those samples with elevated CSF l-lactate, the WCC was elevated in only 5 (and neutrophils in none), and the frequency of CSF l-lactate elevation did not differ significantly between samples with and without neutrophil granulocytes (40% [4/10] vs. 35% [13/37]), widely ruling out neutrophil pleocytosis as a major cause of CSF l-lactate elevation in our patients. However, a weak yet significant positive correlation of l-lactate with the CSF WCC was found (*r* = 0.199, *p* < 0.04; *r*^2^ = 0.04, *p* < 0.04; *N* = 109 samples with available data, from 97 patients). l-Lactate levels were also significantly higher in patients who required ventilation at the time of LP or within 1 week before or after LP (median 2.2 vs. 1.75 mmol/l; *p* < 0.00002). Finally, CSF l-lactate levels correlated with QAlb (*r* = 0.307, *p* < 0.003; *r*^2^ = 0.094, *p* < 0.003; *N* = 96 samples with available data, from 87 patients) and, accordingly, also with CSF total protein (*r* = 0.237, *p* < 0.02; *r*^2^ = 0.056, *p* < 0.02; *N* = 106 samples with available data, from 95 patients). The correlation of L-lactate with QAlb was much stronger after exclusion of samples with unusually high QAlb values ≥ 30 × 10^–3^ (*r*^2^ = 0.24; *p* < 0.0001; *N* = 91 samples with available data, from 85 patients) (Fig. 3B).

CSF l-lactate levels were also significantly higher (*p* < 0.002) and CSF l-lactate elevation significantly more frequent (76.5 vs. 17%; *p* < 0.00001) in patients with elevated QAlb than in those with normal QAlb.

### Intrathecal IgG synthesis

In 58/103 (56.3%) samples of group I tested, identical oligoclonal IgG bands in serum and CSF with no additional CSF-restricted bands (the so-called ‘mirror pattern’ or ‘OCB pattern 4’ [21, 22, 35]) were present, a pattern thought to reflect extrathecal immune activation and passive diffusion of peripheral oligoclonal IgG from the serum into the CSF. By contrast, CSF-restricted OCB, indicative of intrathecal IgG synthesis [21, 22, 35], were positive in only 2/103 (1.9%) samples (pattern 3 in both, pattern 2 in none) or 2/96 (2.1%) patients. QIgG, another, albeit less sensitive, marker of intrathecal IgG synthesis, was elevated (8.3; Q_lim_[IgG] 5.6) in just 1/115 (1%) samples or 1/106 (0.9%) patients tested, namely in a patient with CSF-restricted OCB and multiple brain infarctions (Table 3 and Fig. 1G). In this patient, the intrathecal IgG fraction was 32%, corroborating the positive OCB result, which corresponded to an absolute amount of intrathecally produced IgG of 38 mg/l. In the second patient with CSF-restricted OCB, QIgG was particularly low (1.86) and did not exceed Q_lim_(IgG). However, low amounts of intrathecal IgG may in fact be detectable only by isoelectric focusing but not by QIgG determination; of note this patient was one of only two with elevated QIgM (see below). In the first patient, no pre- or coexisting condition known to cause OCB positivity has been detected as of last follow-up; the second patient had been previously diagnosed with mild dementia of unknown cause. Pattern 5, indicating monoclonal gammopathy, was present in 1/103 samples (1%), obtained from a patient with punctate multiple ischemia on brain MRI, bilateral visual impairment, decreased vigilance, and capillary leak syndrome. Altogether, intrathecal IgG synthesis was very rare and even below the rate reported in healthy central European volunteers [36].

**Table 3:** Frequency of intrathecal IgG synthesis, oligoclonal IgG patterns, IgG CSF/serum ratios, intrathecal IgG fractions, absolute amount of locally produced IgG, and absolute IgG concentrations in the CSF and serum in group I

	Units	Total	≤ 14 d	> 14 d	Acute B/SC subgroup	Acute PN/C/H subgroup
Intrathecal IgG synthesis						
OCB positive or IgG-IF ≥ 10%	Samples	2/103 (1.9%)	1/71 (1.4%)	0/21 (0%)	1/57 (1.8%)	0/13 (0%)
OCB positive	Samples	2/103 (1.9%)	1/71 (1.4%)	0/21 (0%)	1/56 (1.8%)	0/12 (0%)
OCB pattern 1	Samples	42/103 (40.8%)	33/71 (46.5%)	6/21 (28.6%)	23/56 (41.1%)	9/12 (75%)
OCB pattern 2	Samples	0/103 (0%)	0/71 (0%)	0/21 (0%)	0/56 (0%)	0/12 (0%)
OCB pattern 3	Samples	2/103 (1.9%)	1/71 (1.4%)	0/21 (0%)	1/56 (1.8%)	0/12 (0%)
OCB pattern 4	Samples	58/103 (56.3%)	36/71 (50.7%)	15/21 (71.4%)	31/56 (55.4%)	3/12 (25%)
OCB pattern 5	Samples	1/103 (1%)	1/71 (1.4%)	0/21 (0%)	1/56 (1.8%)	0/12 (0%)
OCB pattern 2 or 3	Samples	2/103 (1.9%)	1/71 (1.4%)	0/21 (0%)	1/56 (1.8%)	0/12 (0%)
OCB pattern 3 or 4	Samples	60/103 (58.3%)	37/71 (52.1%)	15/21 (71.4%)	32/56 (57.1%)	3/12 (25%)
OCB pattern 1, 4, or 5	Samples	101/103 (98.1%)	70/71 (98.6%)	21/21 (100%)	32/56 (98.2%)	3/12 (100%)
QIgG > Qlim(IgG)	Samples	1/115 (1%)	1/81 (1%)	0/21 (0%)	1/64 (1.6%)	0/16 (0%)
QIgG, all LPs	–	3.7 (0.9–22.4;115)	3.8 (1–22.4;81)	4.1 (1.2–6.8;21)	3.85 (1.54–22.4;64)	3.7 (0.96–12.1;16)
QIgG, if positive	–	8.3 (8.3–8.3;1)	8.3 (8.3–8.3;1)	n.a. (n.a.;0)	8.25 (8.25–8.25;1)	n.a. (n.a.;0)
IgG-IF, all LPs	% IgG(CSF)	0 (0–32.2;115)	0 (0–32.2;81)	0 (0–0;21)	0 (0–32.2;64)	0 (0–0;16)
IgG-IF, QIgG positives	% IgG(CSF)	32.2 (32.2–32.2;1)	32.2 (32.2–32.2;1)	n.a. (n.a.;0)	32.2 (32.2–32.2;1)	n.a. (n.a.;0)
IgG-IF, > 10%	Samples	1/115 (0.9%)	1/81 (1.2%)	0/21 (0%)	1/64 (1.6%)	0/16 (0%)
IgG-loc, all LPs	mg/l	0 (0–38;115)	0 (0–38;81)	0 (0–0;21)	0 (0–38;64)	n.a. (n.a.; 0)
IgG-loc, QIgG positives	mg/l	38 (38–38;1)	38 (38–38;1)	n.a. (n.a.;0)	38 (38–38;1)	n.a. (n.a.;0)
IgG CSF, all LPs	mg/l	40.4 (6.2–239;115)	41.7 (8–239;81)	40.4 (9.6–69;21)	43.75 (12–239;64)	34.95 (8–135;16)
IgG CSF, QIgG positives	mg/l	118 (118–118;1)	118 (118–118;1)	n.a. (n.a.;0)	118 (118–118;1)	n.a. (n.a.;0)
IgG serum, all LPs	g/l	11.3 (3.4–28.8;120)	11.3 (3.4–21.1;81)	10.7 (6.1–18.2;22)	11.45 (3.4–21.1;64)	11 (6.34–18.2;16)
IgG serum, QIgG positives	g/l	14.3 (14.3–14.3;1)	14.3 (14.3–14.3;1)	n.a. (n.a.;0)	14.3 (14.3–14.3;1)	n.a. (n.a.;0)
IgG serum, elevated	Samples	12/120 (10%)	5/81 (6.2%)	1/22 (4.5%)	4/64 (6.3%)	1/16 (6.3%)
Link index, all	Samples	1/114 (1%)	1/80 (1%)	0/21 (0%)	1/63 (1.6%)	0/16 (0%)
Link index, if positive	Index	1.1 (1.1–1.1;1)	1.1 (1.1–1.1;1)	n.a. (n.a.;0)	1.1 (1.1–1.1;1)	n.a. (n.a.;0)

Quotients, indices, concentrations, and fractions are given as medians (with ranges and sample numbers in brackets). *B/SC* brain/spinal cord, *PN/CN/H* peripheral nerve/cranial nerve/headache only, *OCB* oligoclonal IgG bands, *QIgG/A/M* CSF/serum IgG/A/M ratio, *IgG/A/M IF* intrathecally produced IgG/IgA/IgM fraction; *IgG/A/M loc* locally (intrathecally) produced IgG/A/M; *LP* lumbar puncture

Unfortunately, CSF SARS-CoV-2-IgG was determined in none of the 2 patients with CSF-restricted OCB; a SARS-CoV-2 CSF PCR was performed in one and was negative. In the only patient with pattern 5 OCB, no SARS-CoV-2-IgG was found in the CSF and SARS-CoV-2 CSF PCR was negative, suggesting that the monoclonal band observed in this case was likely unrelated to SARS-CoV-2 infection.

The frequency of pattern 4 (‘mirror pattern’, i.e. identical OCB in serum and CSF) [21, 22, 35] was higher in samples obtained > 14 days after onset of neurological symptoms (71.4%) than in samples obtained earlier, possibly reflecting the increase in systemic SARS-CoV-2 antibody production as well as general immune activation during the first few weeks after infection. In contrast, the proportion of patients with pattern 1 (no OCB), declined over time (≤14 days: 46.5%, *N* = 71; > 14 days: 28.6%, N = 21), although the difference did not reach statistical significance.

Of note, CSF IgG concentrations exceeded the upper reference limit of 40 mg/l in 50/100 (50%) OCB-negative samples tested (Table 3). However, this must not be mistaken for evidence of an intrathecal immune response in COVID-19. In none of these cases, QIgG was elevated, indicating that the increased CSF concentrations were caused by passive transfer of IgG rather and not by intrathecal synthesis. Indeed, QAlb, indicating a leaky BCB, was increased in 80% (40/50) of these patients, but in only 20% (10/50) of those with normal CSF IgG values and available data. Moreover, CSF IgG values were strongly dependent on QAlb, both among patients with elevated QAlb (*r* = 0.863, *p* < 0.00001; *r*^2^ = 0.745, *p* < 0.00001; *N* = 57 samples with available data, from 54 patients) and in the total cohort (*r* = 0.871, *p* < 0.00001; *r*^2^ = 0.759, *p* < 0.00001; *N* = 115 samples with available data, from 106 patients) (Fig. 3C, D). QAlb was predictive of CSF IgG in those exceeding the reference limit of 40 mg/l (*r* = 0.83, *p* < 0.00001; *r*^2^ = 0.689, *p* < 0.00001; *N* = 58 samples with available data, from 53 patients) but serum IgG levels were not, suggesting that elevated CSF IgG levels were mainly driven by BCB dysfunction.

Neither CSF IgG levels (43.75 mg/l, *N* = 64 vs. 34.95 mg/l, *N* = 16) nor median IgG CSF/serum ratios (3.85, *N* = 64 vs. 3.7, *N* = 16) differed significantly between the acute ‘B/SC subgroup’ and the ‘PN/CN/H subgroup’. Similarly, no significant difference in median CSF IgG levels (41.7 mg/l, *N* = 81 vs. 40.4 mg/l, *N* = 21) or median QIgG values (3.8, *N* = 81 vs. 4.1, *N* = 21) was observed between samples taken during the first 14 days after onset of neurological symptoms and those taken later.

**Fig. 4:**
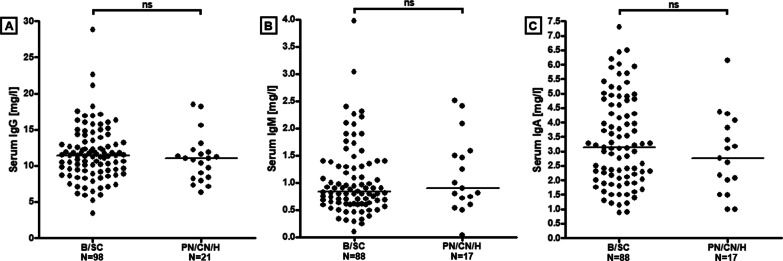
No statistically significant differences in serum IgG (**A**), IgM (**B**) and IgA (**C**) levels between the ‘B/SC subgroup’ and the ‘PN/CN/H subgroup’. *B/SC* brain spinal cord, *N* number of samples. *PN/CN/H* peripheral nerve/cranial nerve/headache only

Serum IgG levels were elevated (> 16 g/l) in 12 samples (10%; from 11 patients) out of 120 samples tested. Six of these patients were tested for serum SARS-CoV-2-IgG and 5/6 were positive. Median serum IgG concentrations did not differ significantly between acute samples (≤ 14 days after onset of neurological symptoms) and samples obtained later (Table 3) nor between the ‘B/SC subgroup’ and the ‘PN/CN/H subgroup’ (Table 3 and Fig. 4A). Median serum IgG levels were higher in samples from patients with positive serum SARS-CoV-2-IgG than in the few samples from SARS-CoV-2-IgG-seronegative patients (11.25 g/l [N = 46] vs. 8.30 g/l [*N* = 7]); however, the difference did not reach statistical significance.

### Intrathecal IgA synthesis

QIgA was increased in just 1/101 (1%) samples tested (Table 4 and Fig. 1H), obtained from a patient with disturbed consciousness (QIgA = 6.6). However, the intrathecal fraction (3.2%) and the absolute amount of intrathecally produced IgA (0.8 mg/l) were very low in this case, leaving the possibility of a false-positive result.

**Table 4:** Frequency of intrathecal IgM and IgA synthesis, IgM and IgA CSF/serum ratios, intrathecal IgM and IgA fractions, amount of locally produced IgM and IgA, and absolute IgM and IgA concentrations in the CSF and serum in group I

	Units	Total	≤ 14 d	> 14 d	Acute B/SC subgroup	Acute PN/CN/H subgroup
Intrathecal IgA synthesis						
QIgA > Qlim(IgA)	Samples	1/101 (1%)	0/72 (0%)	1/19 (5%)	0/59 (0%)	0/12 (0%)
QIgA, all LPs	–	2.1 (0.4–17.6;101)	2.1 (0.6–17.6;72)	2.4 (0.7–6.6;19)	2.19 (0.56–17.6;59)	1.78 (0.69–7;12)
QIgA, if positive	–	6.6 (6.6–6.6;1)	n.a. (n.a.;0)	6.6 (6.6–6.6;1)	n.a. (n.a.;0)	n.a. (n.a.;0)
IgA-IF, all LPs	% IgA(CSF)	0 (0–3.2;101)	0 (0–0;72)	0 (0–3.2;19)	0 (0–0;59)	0 (0–0;12)
IgA-IF, QIgA positives	% IgA(CSF)	3.2 (3.2–3.2;1)	n.a. (n.a.;0)	3.2 (3.2–3.2;1)	n.a. (n.a.;0)	n.a. (n.a.;0)
IgA-IF, > 10%	Samples	0/101 (0%)	0/72 (0%)	0/19 (0%)	0/59 (0%)	0/12 (0%)
IgA-loc, all LPs	mg/l	0 (0–0.8;101)	0 (0–0;72)	0 (0–0.8;19)	0 (0–0;59)	0 (0–0;12)
IgA-loc, QIgA positives	mg/l	0.8 (0.8–0.8;1)	n.a. (n.a.;0)	0.8 (0.8–0.8;1)	n.a. (n.a.;0)	n.a. (n.a.;0)
IgA CSF	mg/l	6.04 (0.9–56.3;101)	6.38 (1.02–56.3;72)	7.01 (1–26.1;19)	7.01 (1.29–56.3;59)	5.22 (1.02–28.5;12)
IgA serum	g/l	3.11 (0.87–7.29;106)	3.1 (0.87–7.29;72)	3.16 (0.9–6.5;20)	3.1 (0.87–7.29;59)	2.89 (1–6.14;12)
IgA serum, elevated	Samples	31/106 (29%)	20/72 (27.8%)	7/20 (35%)	16/59 (27%)	4/12 (33%)
Intrathecal IgM synthesis						
QIgM > Qlim(IgM)	Samples	4/100 (4%)	2/72 (3%)	0/19 (0%)	2/58 (3%)	0/13 (0%)
QIgM, all LPs	–	0.5 (0.1–20.2;100)	0.5 (0.1–20.2;72)	0.4 (0.1–1.6;19)	0.56 (0.11–20.2;58)	0.5 (0.16–4.96;13)
QIgM, if positive	–	4.2 (1–20.2;4)	12.6 (4.9–20.2;2)	n.a	12.56 (4.9–20.2;2)	n.a. (n.a.;0)
IgM-IF, all LPs	% IgM(CSF)	0 (0–49.2;100)	0 (0–12.1;72)	0 (0–0;19)	0 (0–12.1;58)	0 (0–0;13)
IgM-IF, QIgM positives	% IgM(CSF)	21.7 (11.6–49.2;4)	11.8 (11.6–12.1;2)	n.a. (n.a.;0)	11.8 (11.6–12.1;2)	n.a. (n.a.;0)
IgM-IF, > 10%	Samples	4/100 (4%)	2/72 (2.8%)	0/19 (0%)	2/58 (3.4%)	0/13 (0%)
IgM-loc, all LPs	mg/l	0 (0–2.1;100)	0 (0–2.1;72)	0 (0–0;19)	0 (0–2.1;58)	0 (0–0;13)
IgM-loc, QIgM positives	mg/l	0.88 (0.46–2.11;4)	1.32 (0.53–2.11;2)	n.a	1.3 (0.5–2.1;2)	n.a. (n.a.;0)
IgM CSF	mg/l	0.49 (0.1–18.2;100)	0.5 (0.1–18.2;72)	0.49 (0.2–2.54;19)	0.5 (0.14–18.2;58)	0.44 (0.1–6.2;13)
IgM serum	g/l	0.86 (0.04–3.98;106)	0.9 (0.1–3.98;73)	0.81 (0.31–2.1;19)	0.9 (0.1–3.98;59)	0.9 (0.5–2.51;13)
IgM serum, elevated	Samples	6/106 (6%)	5/73 (6.8%)	0/19 (0%)	4/59 (7%)	1/13 (8%)

Quotients, concentrations, and fractions are given as medians (with ranges and samples numbes in brackets). *B/SC* brain spinal cord, *PN/CN/H* peripheral nerve/cranial nerve/headache only, *QIgG/A/M* CSF/serum IgG/A/M ratio, *IgG/A/M IF* intrathecally produced IgG/IgA/IgM fraction, *IgG/A/M* *loc* locally (intrathecally) produced IgG/A/M, *LP* lumbar puncture

Notably, IgA serum levels were elevated (> 4 g/l) in 31 samples (29%; from 29 patients) out of 106 samples  tested in group I, ranging between 4.1 and 7.3 g/l (median 5). Of these samples, 8 were tested for SARS-CoV-2-IgA and 6/8 were positive. Median CSF and serum IgA concentrations and CSF/serum ratios did not differ significantly between samples obtained ≤ 14 days since onset of the neurological symptoms and samples obtained later (Table 4) nor between the ‘B/SC subgroup’ and the ‘PN/CN/H subgroup’ (Table 4 and Fig. 4C), although median values were all slightly higher in the former subgroup.

### Intrathecal IgM synthesis

QIgM was increased in only 4/100 (4%) samples (1–20.2) tested from 3 patients in group I, including the abovementioned patient with pre-existing mild dementia and CSF-restricted OCB but normal QIgG. In these 4 samples, the median fraction of intrathecally produced IgM was 22% (12, 12, 31 and 49%, respectively) and thus > 10% (considered indicative of true-positive intrathecal synthesis [20]) in all cases. The absolute amount of intrathecally produced IgM in these samples was 0.46–2.11 mg/l, respectively (Table 4). Erythrocyte counts were < 2500/µl in all four cases (0, 0, "< 100", "< 1000"), which argues against a major effect of blood contamination on QIgM levels [37].

Serum IgM levels were elevated (> 2.3 g/l) in 6 samples (6%; from 6 patients) out of 106 samples tested (none of the six samples was tested for SARS-CoV-2-IgM). Median CSF and serum IgM concentrations and CSF/serum ratios did not differ significantly between samples obtained ≤ 14 days after onset of neurological symptoms and samples obtained later (Table 4) and also not between the ‘B/SC subgroup’ and the ‘PN/CN/H subgroup’ (Table 4 and Fig. 4B).

### Immunoglobulin (Ig) class patterns

None out of 99 (0%) samples from 90 SARS-CoV-2-positive patients tested in group I exhibited the three-class-reaction (as defined by elevation of QIgG, QIgM and QIgA) or two-class-reaction (defined by either positive QIgG and QIgM, positive QIgM and QIgA, or positive QIgG and QIgA), based on QIg > Q_lim_(Ig) (Table 5), often seen in viral and bacterial infections of the CNS.

**Table 5:** Immunoglobulin class response patterns (ICRPs) in group I

	Units	Total	≤ 14 d	> 14 d	Acute B/SC subgroup	Acute PN/CN/H subgroup
*a. Based on QIg* > *Qlim(Ig)*						
3-class reaction	Samples	0/99 (0%)	0/71 (0%)	0/19 (0%)	0/58 (0%)	0/12 (0%)
2-class reaction	Samples	0/99 (0%)	0/71 (0%)	0/19 (0%)	0/58 (0%)	0/12 (0%)
IgG + IgM	Samples	0/99 (0%)	0/71 (0%)	0/19 (0%)		
IgG + IgA	Samples	0/99 (0%)	0/71 (0%)	0/19 (0%)		
IgM + IgA	Samples	0/99 (0%)	0/71 (0%)	0/19 (0%)		
1-class reaction	Samples	6/99 (6.1%)	3/71 (4.2%)	1/19 (5.3%)	3/58 (5.2%)	0/12 (0%)
Only IgG	Samples	1/99 (1%)	1/71 (1.4%)	0/19 (0%)		
Only IgM	Samples	4/99 (4%)	2/71 (2.8%)	0/19 (0%)		
Only IgA	Samples	1/99 (1%)	0/71 (0%)	1/19 (5.3%)		
*b. Based on Ig-IF* > *10%*						
3-class reaction	Samples	0/99 (0%)	0/71 (0%)	0/19 (0%)	0/58 (0%)	0/12 (0%)
2-class reaction	Samples	0/99 (0%)	0/71 (0%)	0/19 (0%)	0/58 (0%)	0/12 (0%)
IgG + IgM	Samples	0/99 (0%)	0/71 (0%)	0/19 (0%)		
IgG + IgA	Samples	0/99 (0%)	0/71 (0%)	0/19 (0%)		
IgM + IgA	Samples	0/99 (0%)	0/71 (0%)	0/19 (0%)		
1-class reaction	Samples	5/99 (5.1%)	3/71 (4.2%)	0/19 (0%)	3/58 (5.2%)	0/12 (0%)
Only IgG	Samples	1/99 (1%)	1/71 (1.4%)	0/19 (0%)		
Only IgM	Samples	4/99 (4%)	2/71 (2.8%)	0/19 (0%)		
Only IgA	Samples	0/99 (0%)	0/71 (0%)	0/19 (0%)		

ICRPs have been shown to be of differential diagnostic relevance in various neurological disorders [13, 14, 19, 20, 35]

Intrathecal Ig synthesis was restricted to one immunoglobulin class in 6/99 (6.1%) samples with available data (IgG in 1; IgM in 4; IgA in 1) from 5 patients based on Ig CSF/serum ratios and in 5/99 (5.1%) samples based on Ig-IF > 10% (Table 5).

In one (IgM-IF 32%) out of 5 patients with intrathecal IgM and/or IgA synthesis but no quantitative evidence of intrathecal IgG synthesis, at least qualitative evidence for intrathecal IgG synthesis (i.e., CSF-restricted IgG OCB) was detectable.

### SARS-CoV-2 antibody indices

In total, 70 individual SARS-CoV-2-IgG, -IgM or -IgA AI tests were performed in group I. This included 46 individual SARS-CoV-2-IgG AI determinations (13 × AIs for nucleocapsid [N]; 18 × spike protein subunit 1 [S1]; 13 × spike protein subunit 2 [S2]; 1 × mixture of N, S1 and S2; 1 × antigens not documented) in 20 samples from 19 patients. Remarkably, only 1 sample yielded an unequivocally positive result (AI > 1.5), indicating that COVID-19 with neurological involvement is not associated with substantial intrathecal IgG synthesis against SARS-CoV-2 in the majority of cases, at least not in the acute stage shortly after onset of the neurological symptoms (the median interval for all patients tested was 5 days; range 1–49), i.e., at the time when LP is mostly performed.

In that sample, elevated IgG AI for the SARS-CoV-2-N (4.8), the SARS-CoV-2-S1 (6.9) and the SARS-CoV-2-S2 (2.2) antigens were found, and, in addition, positive SARS-CoV-2-IgM AI (N: 2.4, S1: 6.5, S2: 5) and positive SARS-CoV-2-IgA AI (N: 1.8, S2: 2.8; S1: normal [1.25]). By contrast, total IgG OCB were negative, suggesting that intrathecally produced SARS-CoV-2-IgG contributed relatively little to the total IgG concentration in the CNS. Of note, the sample exhibited an unusually high WCC (247 cells/µl).

In a further patient—one of only two other patients in this cohort with a WCC > 50 (510 cells/µl) –, the SARS-CoV-2-N-IgG AI was 1.35. According to current guidelines [35], AI values > 1.3 should be considered positive if substantially higher than other AIs in the same sample, based on the assumption that immunoglobulin molecules of the same isotype should pass the BBB at the same rate, irrespective of their epitope specificity. Thus, based on the marked difference between the observed IgG-AI of 1.35 for SARS-CoV-2 and the very low VZV-IgG-AI (< 0.5) found in the same paired CSF/serum samples, intrathecal synthesis to SARS-CoV-2 likely occurred also in this case [35]. The SARS-CoV-2-IgA AI and the SARS-CoV-2-IgM AI were not tested in this patient.

Both patients belonged to the ‘B/SC subgroup’. In both samples QAlb was elevated (QAlb = 35.6 × 10^–3^ and 7.4 × 10^–3^, respectively).

SARS-CoV-2-IgM AI and SARS-CoV-2-IgA AI were tested in three additional patients (all with a normal SARS-CoV-2-IgG AI) but were elevated in none (Table 6).

**Table 6:** Antibody indices, CSF antibody concentrations and CSF PCR results for SARS-CoV-2, numerous other viruses, including herpes simplex virus (HSV), varicella zoster virus (VZV), cytomegalovirus (CMV), Epstein Barr virus (EBV), human herpes virus 6 (HHV6), measles virus (M), and rubella virus (R), and *Borrelia burgdorferi* (BB) in group I

	Units	Total cohort
Positive antibody indices		
AI SARS-CoV-2, IgG	Samples	2/20 (10%)
	Patients	2/19 (10.5%)
AI SARS-CoV-2, IgM	Samples	1/4 (25%)
	Patients	1/4 (25%)
AI SARS-CoV-2, IgA	Samples	1/4 (25%)
	Patients	1/4 (25%)
AI HSV, IgG	Samples	1/22 (4.5%)
AI EBV, IgG	Samples	0/4 (0%)
AI CMV, IgG	Samples	0/11 (0%)
AI B. burgdorferi, IgG	Samples	0/21 (0%)
AI B. burgdorferi, IgM	Samples	1/18 (5.6%)
AI measles virus (M), IgG	Samples	0/1 (0%)
AI rubella virus (R), IgG	Samples	0/1 (0%)
AI varicella zoster virus (Z), IgG	Samples	1/20 (5%)
MRZ reaction (M + R, M + Z, R + Z, or M + R + Z)	Samples	0/1 (0%)
	Patients	0/1 (0%)
Elevated CSF antibody levels		
SARS-CoV-2, IgG	Samples	20/29 (69%)
	Patients	20/28 (71.4%)
SARS-CoV-2, IgM	Samples	1/4 (25%)
	Patients	1/4 (25%)
SARS-CoV-2, IgA	Samples	1/4 (25%)
	Patients	1/4 (25%)
Positive CSF PCR		
SARS-CoV-2	Samples	0/76 (0%)
	Patients	0/75 (0%)
HSV	Samples	0/59 (0%)
VZV	Samples	1/21 (4.8%)
CMV	Samples	0/20 (0%)
EBV	Samples	0/6 (0%)
HHV6	Samples	0/5 (0%)
Neurotropic viruses (panel)	Samples	1/2 (50%)

Importantly, the median interval between onset of the neurological symptoms and AI testing was not longer in the AI-positive subgroup (4 days; range 4–4) than in the AI-negative subgroup (5.5 days; range 1–49).

As a limitation, different assays were used to determine SARS-CoV-2 AI at different centers. While in-house assays were used by one center (University Hospital Zurich, Switzerland) [38], commercial assays manufactured by Euroimmun (Lübeck, Germany) and Generic Assays (Berlin, Germany) were adapted and used for CSF analysis by others. No officially approved SARS-CoV-2-AI assays are currently available in Europe.

### CSF SARS-CoV-2-IgG

Absolute SARS-CoV-2-IgG concentrations in the CSF exceeded the assay-specific upper reference levels in 20/29 (69%) samples tested in group I (Table 6), including the two SARS-CoV-2-IgG-AI-positive samples. 13 samples were tested separately for CSF IgG against N, S1 and S2; the 8 positives among these samples all reacted against all three antigens (and the 5fff negative samples were negative for SARS-CoV-2-IgG against all three antigens).

In addition, SARS-CoV-2 CSF IgM and IgA levels were elevated in 1/4 (25%) and 1/4 (25%) samples tested, respectively, namely in the patient with a high WCC (247 cells/µl) and a positive SARS-CoV-2-IgG AI mentioned above.

### SARS-CoV-2 CSF PCR

Of particular note, CSF PCR for SARS-CoV-2 was negative in 76/76 (100%) samples from 75 patients with neurological symptoms tested in group I, including those with an increased SARS-CoV-2-IgG (and -IgM) AI, all of those with a WCC > 50, and 63/63 from patients with B/SC involvement (with neurological symptoms classified as “severe” in 41 of them). The median time between neurological onset and PCR was just 5 days (percentile range [0.1–0.9] 1–25). However, 26 samples were taken 7 or more days and 15 of these 14 or more days after neurological onset. PCR was negative in 69 initial CSF samples and 6 CSF samples obtained at follow-up LP (in 6 different patients). CNS tissue PCR from biopsy or autopsy samples was performed in none of the patients.

### CSF PCR for other viruses

Results from 113 further PCR tests in 32 samples from 30 patients were available for analysis (59 × HSV, 20 × CMV, 6 × EBV, 5 × HHV6, and 2 × panel PCR neurotropic viruses) (Table 6). In one patient, viral metagenome analyses were weakly positive for pegivirus. In another one, CSF VZV PCR was low positive with 150 copies/ml, but no diagnosis of VZV encephalitis was made (6 cells/µl; final diagnosis: GBS). All other tests were negative.

### MRZ reaction

Measles virus (M), rubella virus (R), and varicella zoster virus (Z) IgG AI results were available for only a small number of samples (N = 22) in group I. Moreover, all three AI were tested only in 1 sample and two AI in 0 samples. A positive Z-AI was found in 1/20 (5%) samples (AI = 2.2, cut-off 1.5; negative panel PCR for neurotropic viruses and negative EBV PCR; hyposmia, severe COVID-19 requiring mechanical ventilation, pre-existing rheumatoid arthritis; serum SARS-CoV-2-IgG positive, SARS-CoV-2-IgG-AI not determined), a positive M-AI in 0/1 (0%), and a positive R-AI in 0/1 (0%). A positive MRZ reaction, as defined by the presence of a positive IgG AI for at least two of its three constituents M, R and Z (i.e. by any of the following combinations: MR, MZ, RZ, or MRZ), which is detectable in around 63% of cases in MS [39], was absent in the only patient with available data (Table 6).

### Other antibody indices

A total of 76 further AI tests were conducted in 40 samples in group I. A positive IgG AI (1.7; cut-off 1.5) for herpes simplex virus (HSV) was found in 1/22 (4.5%) samples tested. While symptoms were generally compatible with HSVE in this patient (acute disorientation, personality changes, encephalopathy), HSV-PCR was negative and no CSF pleocytosis present; in consequence, no diagnosis of HSVE was made by the then treating physicians. SARS-CoV-2-AI testing was not done in this case. None of the patients tested had a positive IgG-AI for *Borrelia burgdorferi* (BB), CMV, or EBV; one patient exhibited a borderline positive (1.6; cut-off 1.5) IgM-AIfor BB (Table 6).

### Anti-neuronal and anti-glial antibodies

Over the past few decades, a multitude of anti-neural autoantibodies have been identified in patients with autoimmune encephalitis, myelitis, or polyneuropathy, some of which are considered to be directly pathogenic, and a para- or postinfectious etiology has been suggested for some of these reactivities [40, 41]. Not all centers routinely test for anti-neural antibodies in patients with neurological symptoms of uncertain cause, and antibody panels differ among centers. In this cohort, 54 samples from 48 patients in group I were tested for anti-neuronal, anti-glial and—in a few cases—anti-muscle autoantibodies. Overall, 1509 individual autoantibody results were documented, comprising 743 CSF tests and 766 serum tests. This included 716 CSF and 723 serum results from the ‘B/SC subgroup’. The panel of established autoantibody markers of encephalitis and/or peripheral nerve disease tested included anti-NMDA-R (*N*-methyl-D-aspartate receptor), anti-AMPA1/2-R (α-amino-3-hydroxy-5-methyl-4-isoxazolepropionic acid receptor), anti-GABA-B1/2-R (G-protein coupled receptors B1/2 for gamma-aminobutyric acid), anti-LG1 (Leucine-rich, glioma inactivated protein 1), anti-CASPR (contactin-associated protein-like 2), anti-DPPX (dipeptidyl-peptidase-like protein-6), anti-mGluR5 (metabotropic glutamate receptor 5), anti-glycine receptor, anti-IgLON-5 (IgLON family member 5), anti-dopamin-2 receptor, anti-Hu, anti-Yo, anti-Ri, anti-Ma2/Ta, anti-Ma1, anti-Tr/DNER (Delta and Notch-like epidermal growth factor-related receptor), anti-recoverin, anti-GAD65 (glutamic acid decarboxylase 65), anti-amphiphysin, anti-Zic4 (Zic family member 4), anti-SOX1 (SRY-box transcription factor 1), anti-nicotinergic (i.e. ganglionic) acetylcholine receptors, anti-MOG (myelin oligodendrocyte glycoprotein), anti-AQP4 (aquaporin-4) [42, 43], and anti-sulfatide and anti-ganglioside antibodies ("ganglioside Abs", GM1-IgG/IgM, GM2-IgG/IgM, GM3-IgG/IgM, GM4-IgG/IgM, GQ1b-IgG/IgM, GD1a-IgG/IgM, GD1b-IgG/IgM, GD1c-IgG/IgM, GT1a-IgG/IgM, GT1b-IgG/IgM); in a few patients, anti-muscle autoantibodies were tested in addition (skeletal muscle sections, titin). Furthermore, 34 CSF and 62 serum samples were tested for so-far unknown anti-neural autoantibodies in group I by indirect immunofluorescence (IIF) using cerebellum, autonomic nervous system and/or sensory peripheral nerve sections (Euroimmun, Lübeck) as antigenic substrates.

However, in the vast majority of cases, no anti-neuronal, anti-glial or anti-skeletal antibodies were found (Table 7), except for not further characterized “anti-myelin antibodies” in 6/40 serum samples, as detected using peripheral nerve tissue sections. Anti-myelin antibodies, as detected by IIF, are a relatively frequent finding, and may occur even in control subjects; the clinical significance in the patients studied here is unclear; in one case, a follow-up sample was available and was negative. 0/34 CSF samples were positive for myelin antibodies. MOG antibodies were negative in the only 3 serum samples and the only CSF sample tested.

**Table 7:** Anti-neuronal and anti-glial autoantibody findings in group I

	Serum, pos	CSF, pos
Hu-IgG	0/35	0/40
Yo-IgG	0/35	0/40
Ri-IgG	0/35	0/40
CV2/CRMP5-IgG	0/33	0/39
Tr/DNER-IgG	0/35	0/40
Ma2/Ta (PNMA2)-IgG	0/35	0/40
Ma1-IgG	0/5	0/4
Amphiphysin-IgG	0/34	0/39
GAD65-IgG	1/35	1/40^§^
Zic4-IgG	0/15	0/5
SOX1-IgG	0/15	0/5
mGluR5-IgG	0/27	0/34
GlycinR-IgG	0/28	0/35
Dopamin-2-R-IgG	0/27	0/34
IgLON-5-IgG	0/2	0/2
Recoverin-IgG	0/11	0/1
NMDAR-IgG	1/40	0/39
GABA-B-R-IgG	0/36	0/39
AMPA1/2-R (GluA1/GluA2)-IgG	0/35	0/39
DPPX-IgG	0/34	0/38
LGI1-IgG	0/36	0/40
CASPR2-IgG	0/41	0/39
MOG-IgG	0/3	0/1
AQP4-IgG	0/31	0/36
Nicotinergic AChR-IgG	0/1	0/0
IFT cerebellum	6/40^#^	0/34^#^
IFT intestine	0/8	0/0
IFT peripheral nerve	0/8	0/0
"Sulfatide Abs"	0/1	0/0
"Ganglioside Abs"	0/7	0/0
GM1-IgG/IgM	0/6	0/0
GM2-IgG/IgM	0/1	0/0
GM3-IgG/IgM	0/1	0/0
GM4-IgG/IgM	0/1	0/0
GQ1b-IgG/IgM	0/6	0/0
GD1a-IgG/IgM	0/1	0/0
GD1b-IgG/IgM	0/5	0/0
GD1c-IgG/IgM	0/1	0/0
GT1a-IgG/IgM	0/1	0/0
GT1b-IgG/IgM	0/1	0/0
GT1c-IgG/IgM	0/1	0/0
IFT skeletal muscle	1/6	0/0
Titin-IgG	0/7	0/0
Sum	*9/766*	*1/743*

^#^Antimyelin antibodies of unknown specificity. ^§^Only weakly (equivocally) positive. , *Abs* antibodies, *AMPA1/2-R* α-amino-3-hydroxy-5-methyl-4-isoxazolepropionic acid receptor, *AQP4* aquaporin-4, *CASPR2* contactin-associated protein-like 2, *DNER* Delta and Notch-like epidermal growth factor-related receptor, *DPPX* dipeptidyl-peptidase-like protein-6; GABA-B1/2-R G-protein coupled receptors B1/2 for gamma-aminobutyric acid, *GAD65* glutamic acid decarboxylase 65, *GluR5* metabotropic glutamate receptor 5, *IgLON-5* IgLON family member 5, *LG1* Leucine-rich, glioma inactivated protein 1, *MOG* myelin oligodendrocyte glycoprotein, *NMDAR* N-methyl-D-aspartate, *nAChR* nicotinergic (i.e. ganglionic) acetylcholine receptors, *pos* positive, *SOX1* SRY-box transcription factor 1, *Zic4* Zic family member 4

One patient, who had presented with encephalopathy and seizure, was positive for GAD65 serum antibodies, but the evidence was considered insufficient to make a formal diagnosis of autoimmune encephalitis by the treating physicians at last follow-up (no pleocytosis, no intrathecal IgG synthesis, CSF GAD65-IgG only weakly positive). A further patient tested positive for serum NMDAR-IgG antibodies; the patient’s CSF was not tested for NMDAR-IgG. While a diagnosis of NMDAR encephalitis cannot be excluded based on the symptoms documented in this case (downbeat nystagmus, orofacial myoclonus, impaired consciousness and delirium, in addition to hyposmia and dysgeusia), no such diagnosis was made by the treating physicians who favored a diagnosis of hypoxic brain damage. Finally, antibodies to skeletal muscle cells of unknown specificity were found in 1 further patient. All remaining 1499 antibody tests were negative.

The median time between onset of the neurological or non-neurological symptoms and the date of LP in those tested for autoantibodies was 6 days (percentile range [0.1–0.9] 1–27.4) and 18 days (percentile range 4–46), respectively.

### Free kappa and lambda light chains

Free kappa light chains (FLC-kappa) and free lambda light chains (FLC-lambda) (i.e., immunoglobulin light chains not bound to heavy chains), which are produced by B cells during antibody synthesis in excess and subsequently secreted, are determined by some laboratories as a supportive quantitative marker of (especially intrathecal) IgG synthesis. FLC-kappa, and FLC-lambda [21, 44, 45] were determined in 6 patients of group 1 in the serum and also in a matched CSF sample in 4 of them.

**Fig. 5:**
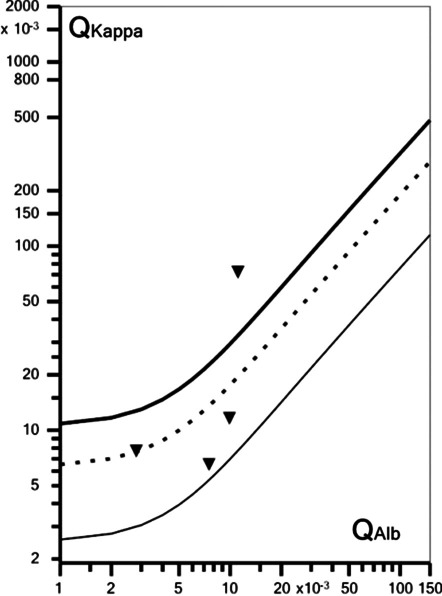
Quotient diagram (‘reibergram’) for free kappa light chains in four patients with SARSV-CoV-2 and neurological symptoms. Graph created using *FLC-K Statistics* v1.02 (Albaum IT Solutions, Möhnesee, Germany)

FLC-kappa concentrations were elevated in 5/6 serum samples (96.45, 40.97, 78.85, 43.51, 137.09 mg/L, respectively; cut-off 19.4) and in 1/4 CSF samples (6.42 mg/l; cut-off 1.96), but in none of the four patients tested was a positive K index ([CSF FLC-kappa/serum FLC-kappa]/[CSF albumin/serum albumin]) found (5.98, 1.1, 0.8 and 2.53, respectively) according to the manufacturer's cut-off (6.35). However, when applying the hyperbolic reference ranges proposed by Reiber et al. (2019) [44] instead of fixed cut-off, intrathecal synthesis of FLC-kappa cannot be ruled out in one case (pattern 4 OCB, BCSF dysfunction, no pleocytosis; CNS subgroup; fatigue, confusion and behavioural disturbances, EEG alterations; intrathecal FLC-kappa fraction 70.6%; locally produced FLC-kappa 4.53 mg/L) (Fig. 5).

Increased FLC-lambda concentrations were present in the serum in 4/6 patients (52.31, 60.91 and 110.3 mg/L, respectively; cut-off 26.3). The L index ([CSF FLC-lambda/serum FLC-lambda]/[CSF albumin/serum albumin]) was normal (cut-off 5.51) in three patients. In the fourth patient, serum and CSF FLC-lambda concentrations did not exceed the upper reference limit, but the L index was elevated (10.61; cut-off 5.51). However, this patient had developed peripheral neuropathy during COVID-19 with signs neither of CNS disease nor of intrathecal total IgG synthesis (normal QIgG, no CSF-restricted OCB) and QAlb was unusually low, rendering a false-positive result at least conceivable.

### Interleukin-6

Interleukin-6 (IL-6) CSF levels were determined in 14 samples from 14 patients (CNS involvement in 13) in group I and were found to be elevated in 11 (79%; 10 × B/SC subgroup; 10 x “severe” neurological disease) according to the manufacturers’ cut-off. The median CSF IL-6 concentration was 10.05 pg/ml (range 1.9- > 300; cut-off 7), with particularly high concentrations (> 20 pg/ml) in 5 samples (45%; 23, 43, 73, > 300, and > 300 pg/ml, respectively; all from the ‘B/SC subgroup’). 

**Fig. 6:**
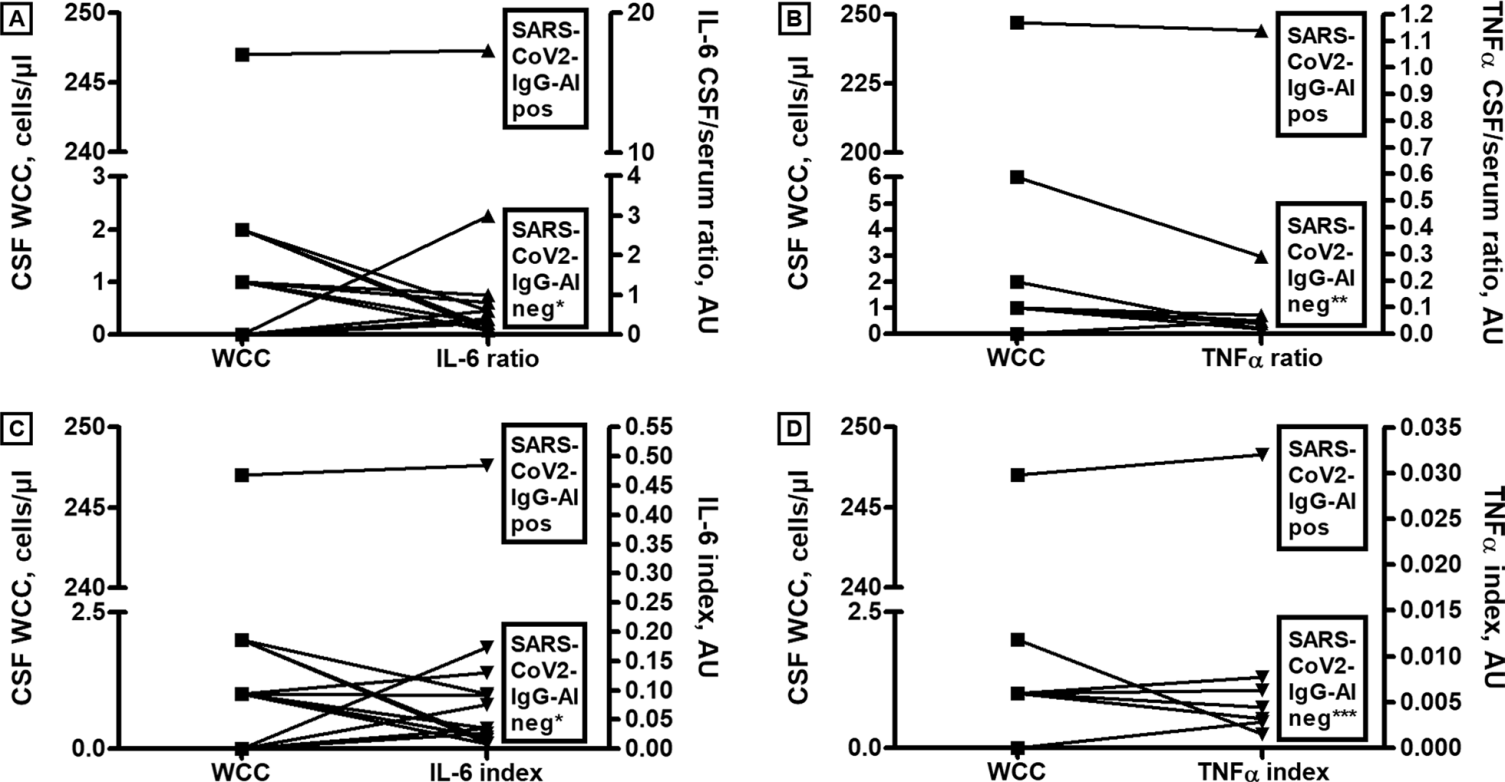
Relationship of CSF WCC, SARS-CoV-2-AI, and IL-6 and TNF-alpha CSF ratios and indices. Data from the same individual patient are connected by a line. *Negative SARS-CoV-2-AI in 10 patients; no SARS-CoV-2-AI data in 2. ** Negative SARS-CoV-2-AI in 4 patients; no SARS-CoV-2-AI data in 3. *** Negative SARS-CoV-2-AI in 4 patients; no AI data in 2. *IL-6* Interleukin-6, *SARS-CoV-2-AI* severe acute respiratory syndrome-coronavirus type 2 antibody index, *TNF-alpha* tumor necrosis factor-alpha, *WCC* white cell count

In a patient with an unusually high WCC (247 cells/µl; lymphomonocytic with small amounts of neutrophils, plasma cells, and activated lymphocytes; CNS involvement) and a positive SARS-CoV-2-AI, IL-6 was determined in both the CSF (> 300 pg/ml) and serum (17.3 pg/ml), revealing a very high IL-6 CSF/serum ratio of > 17 (compared to a ratio of < 2.67 in 95% of German control subjects without neurological disease [46]). IL-6 was not determined in the other patients with marked pleocytosis. An IL-6 CSF/serum ratio > 3 was present in none of 12 further samples tested, none of which showed pleocytosis (median 1 cell/µl; range 0–2) and 10/10 tested had a negative SARS-CoV-2-AI (no data in 2) (Fig. 6).

Increased blood (usually plasma, more rarely serum; same cut-off) IL-6 levels (median 34.3 pg/ml; range 6.4–1075) were noted in 27 (93%; 21 × B/SC subgroup) of 29 samples (from 29 patients) tested, and BCB dysfunction, as indicated by elevated QAlb, was present in 8/10 (80%) samples with elevated CSF IL-6, rendering it possible that IL-6 was partly of peripheral origin in some of these cases.

To take into account a possible effect of BCB dysfunction, we also calculated the IL-6 index (= IL-6 ratio/albumin ratio). Here, the marked difference between the SARS-CoV-2-IgG-AI-positive sample with pleocytosis (247 cells/µl) and the SARS-CoV-2-IgG-AI-negative patients with a normal or almost normal WCC was still present (IL-6 index 0.5 vs. median index of 0.03 in the remainder) (Fig. 6).

**Fig. 7:**
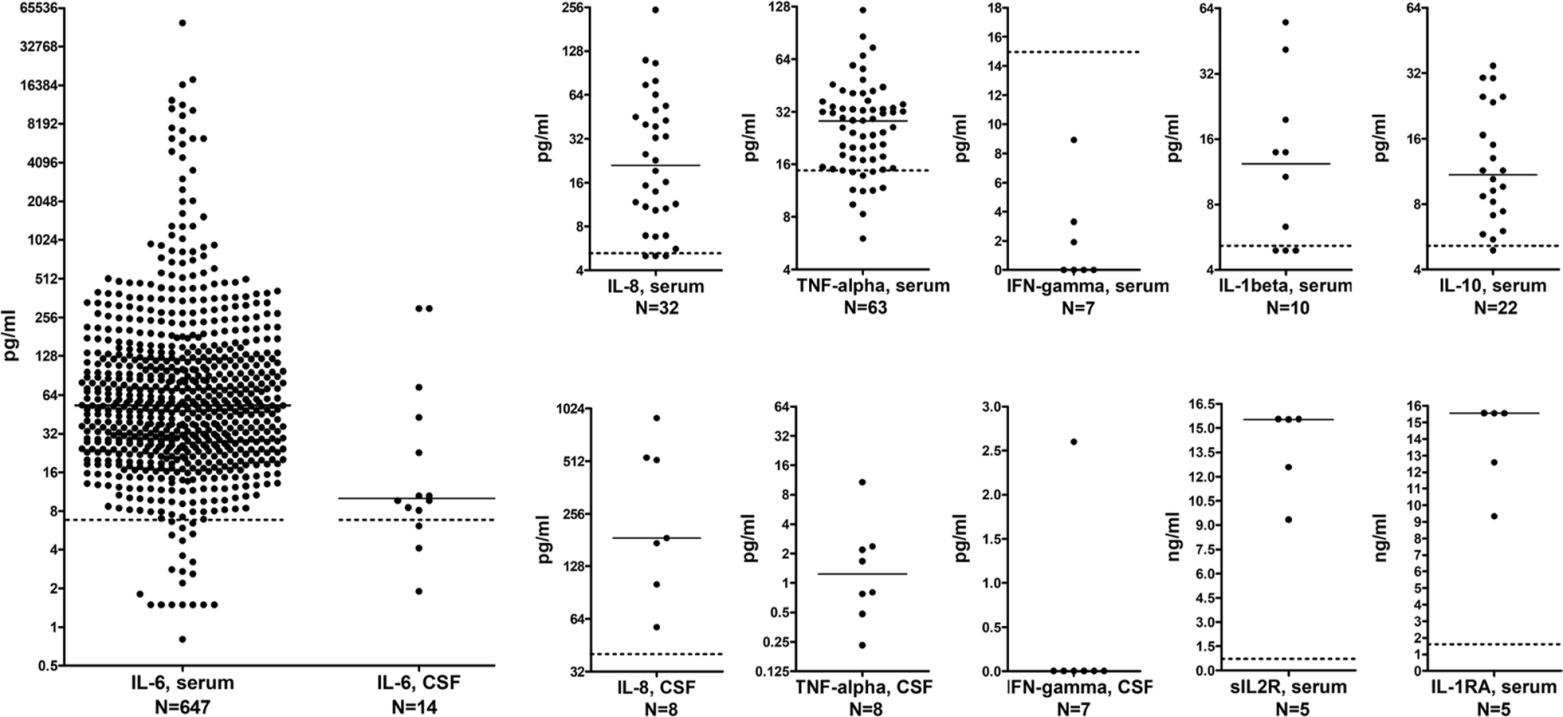
Cytokine and cytokine receptor concentrations in the CSF and serum during hospitalization for COVID-19. *IFN* interferon, *IL* interleukin, *TNF-alpha* tumor necrosis factor-alpha. Dotted lines indicate cut-offs; solid lines indicate medians

**Fig. 8:**
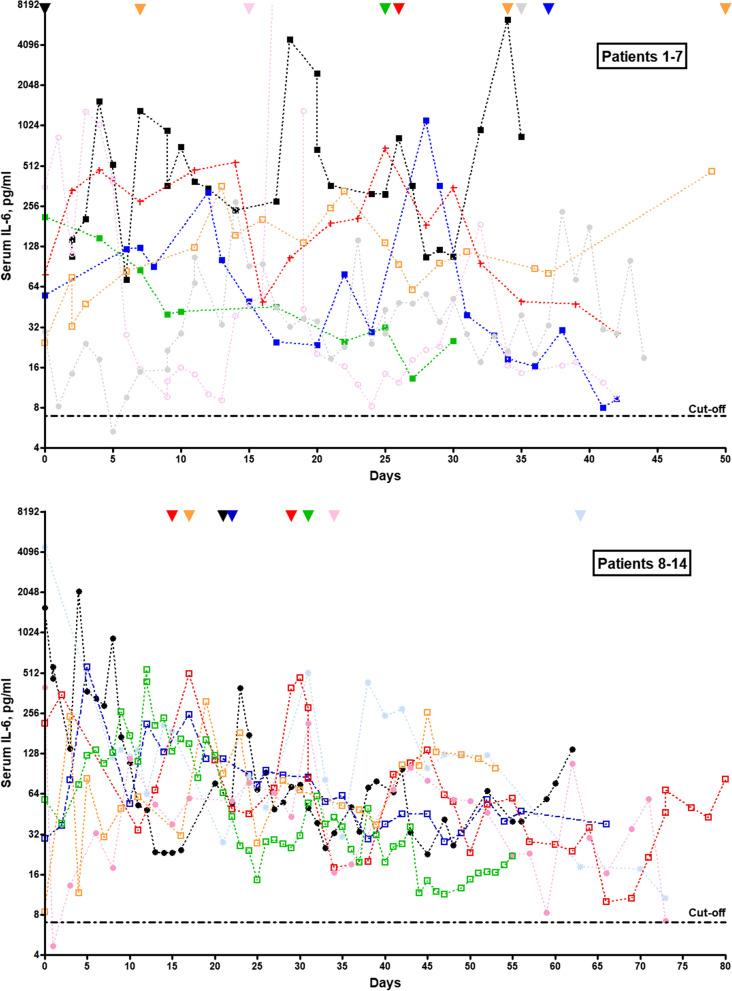
Repeat IL-6 serum measurements over a period of up to 50 (upper panel) or up to 80 (lower panel) days during hospitalization in patients with COVID-19 and neurological complications in 14 patients with available long-term data; each patient is represented by a different colour of symbols and connecting lines. The x-axis indicates days since first IL-6 serum measurement (median 51 days follow-up, range 30-80); if days since the first SARS-CoV-2 PCR-positive swab are considered instead, serum IL-6 was still elevated after at least (last known measurement) 35, 37, 42, 45, 48, 49, 55, 58, 64, 76, 77, 79, 88 and 95 days, respectively, in these patients (median 57 days, range 35-95), partly at high level. The triangles at the top of each panel indicate the time of LP. *IL-6* interleukin-6, *LP* lumbar puncture

In most patients, blood IL-6 levels were repeatedly determined. Overall, 748 samples from 63 patients were tested within a median period of 26.5 days (percentile range [0.1–0.9] 8.3–64.8) around the date of the LP. In total, 723 (96.7%) of these samples exhibited elevated IL-6 levels (median IL-6 concentration 54.45 pg/ml; percentile range 14.99–364) (Fig. 7). In 59/63 (93.7%) patients, IL-6 concentrations were elevated at least once. Of particular note, blood IL-6 levels remained continuously elevated over weeks and months (see Fig. 8 for exemplary data). Serum IL-6 was still elevated at last follow-up (median 28 pg/ml) in 18/19 patients with repeat IL-6 measurements and a follow-up of > 30 days (range 33–80) since first IL-6 measurement and in 12/12 of those with a follow-up of > 45 days (median 33 pg/ml; range 7.2–463) since first measurement, indicating persisting systemic inflammation. From 6 patients follow-up samples obtained over a period of > 60 days were available; serum IL-6 was still elevated in all of these at last follow-up (median 33 pg/ml; range 7.2–138).

Notably, IL-6 was also detectable at high levels in the aqueous humor in the only patient tested because of suspected viral retinitis (1183 pg/ml).

### Interleukin-8

CSF interleukin-8 (IL-8) was elevated in 7/7 (100%; 6 × B/SC subgroup) CSF samples from 7 patients in group I, based on a cut-off of 40 pg/ml derived from a German cohort of patients with normal pressure hydrocephalus (21.40 ± 7.96 pg/ml) [26] (which is in good accordance with the upper reference range found in a recent Chinese healthy control cohort [23.86 ± 17.74 pg/ml] [27] and a cohort of European patients with trigeminal neuralgia [23.1–33.9 pg/ml] [28]). CSF IL-8 concentrations were relatively high, exceeding 50 pg/ml in 7 (100%) and 100 pg/ml in 6 (86%) samples, with a median of 185.7 pg/ml (range 57.4–903.5).

Relevant pleocytosis was present in none of the samples (median 1 cell/µl; range 0–6). Regrettably, none of the 4 samples with high WCC > 100/µl (including all patients with a positive SARS-CoV-2-IgG AI) was tested for CSF IL-8.

Elevated QAlb, indicating BCB dysfunction, was found in 4/6 (67%) samples with elevated CSF IL-8, and plasma IL-8 levels were elevated in 5/8 (63%; 7 × B/SC subgroup) samples tested, suggesting a possible contribution of extrathecally produced IL-8 to CSF IL-8 levels. Whether intrathecal IL-8 synthesis occurred, remains unknown since no well-established cut-off exists. 6/6 samples exhibited a CSF/plasma IL-8 ratio > 1 (median 3.2; range 1.3–4.8). Based on an upper reference limit of 2.7 found in another Central European control cohort that comprised patients with trigeminal neuralgia [28], 3/6 would have exhibited a positive IL-8 ratio.

In 6/7 (86%) samples with elevated CSF IL-8 levels and available data also increased CSF IL-6 levels were found.

Overall, 32 plasma samples from 19 patients in group I were tested for IL-8 during COVID-19 hospitalization. Of these, 29 (90.6%) samples exhibited elevated plasma IL-8 levels (median IL-8 concentration 25 pg/ml; percentile range [0.1–0.9] 6.9–84.88; cut-off 5 pg/ml), and 18/19 (94.7%) patients showed elevated IL-8 concentrations at least once.

### TNF-alpha

The normal range for tumor necrosis factor alpha (TNF-alpha) in the CSF is not well defined (e.g. 0.18 ± 0.16 pg/ml in [29], 3.29 ± 6.61 pg/ml in [30], 6.6 ± 0.5 pg/ml in [31], up to 67 pg/ml in [32], 22.3 ± 9.5 in [33]). CSF TNF-alpha levels were measured in 8 samples from 8 patients in group I and ranged from 0.23 to 10.73 pg/ml (median 1.23 pg/ml). Increased serum TNF-alpha levels were present in 5/10 (50%; 8 × B/SC subgroup) samples according to the manufacturers’ cut-offs (median 14.9 pg/ml; range 6–40.8).

The CSF sample with the highest TNF-alpha level (10.37 pg/ml) came from the abovementioned patient with an unusually high WCC (247 cells/µl), an unusually high IL-6 ratio and index, an elevated SARS-CoV-2-IgG AI, compatible with possible direct CNS infection with SARS-CoV-2, and severe clinical CNS involvement. The corresponding serum sample was positive for TNF-alpha at a concentration of 9.4 pg/ml, resulting in a CSF/serum ratio of 1.14. By contrast, the TNF-alpha CSF/serum ratio was markedly below 1 and thus at least 10 times lower in all (N = 7) further cases with available data (median 0.05; range 0.02–0.29), 6 of them without pleocytosis, and also not beyond the mean TNF-alpha CSF/serum ratio (0.29) reported in patients with low backpain [30].

A marked difference was also found for the TNF-alpha index (= TNF-alpha ratio/albumin ratio), which was 0.032 in the SARS-CoV-2-IgG-AI-positive sample with marked pleocytosis and a positive IL-6 index and 0.0038 (median; range 0.0015–0.008) in the remainder with normal or almost normal WCC (Fig. 6).

Overall, serum or plasma TNF-alpha was determined in 63 samples from 26 patients during hospitalization for COVID-19 and was elevated (median 31.8 pg/ml; percentile range [0.1–0.9] 17.65–56.48; cut-off 15) in 51 (81%) of them. Serum TNF-alpha concentrations were elevated in 21/26 (80.8%) patients at least once.

### Interferon-gamma

Interferon-gamma (IFN-gamma) was detectable in only 1 CSF sample (from the B/SC subgroup) out of 7 samples from 7 patients tested (6 × B/SC subgroup), at a concentration of 2.6 pg/ml. While a generally accepted reference range for CSF IFN-gamma has not been defined, that level is lower than the mean CSF IFN-gamma concentrations in a recent cohort of control patients with non-inflammatory neurological diseases (mean 7.99 ± 2.75 pg/ml) [27], in patients with vertebrogenic low backpain in another cohort (49.93 ± 30.28 pg/ml) [30], and in a cohort of “symptomatic controls” in a further study (3.09 ± 10.64 pg/ml) [34]. Serum IFN-gamma was normal in 7/7 (100%; 6 × B/SC subgroup) serum samples from 7 patients (median 0 pg/ml; range 0–8.9; cut-off 15).

### Interleukin-1beta

Interleukin-1beta (IL-1beta) was determined in 10 plasma samples in group I, and was positive in 3 (30%) samples from 3/9 (33.3%) patients (median 13.9 pg/ml; percentile range 8.94–46.76; normal < 5 pg/ml). IL-1beta secretion by monocytes after lipopolysaccharide (LPS) stimulation for 24 h was measured in 4 patients and was elevated in all of them (316 pg/ml; percentile range 308.8–323.2; normal range 28–204).

### Anti-inflammatory cytokines

Interleukin-10 (IL-10) was elevated (median 10.4 pg/ml; percentile range 5.96–30.32; cut-off 5) in 21/22 (95.5%) serum samples (from 12 patients) obtained during hospitalization for COVID-19 in group I. Overall, 11/12 (91.7%) patients showed increased serum levels at least once. Of note, IL-10 levels were also increased in aqueous humor in one patient (177 pg/ml). Serum concentrations of the anti-inflammatory IL-1 receptor antagonist (IL-1RA), which is induced by IL-10, were markedly increased in 5/5 (100%) serum samples from 2 patients tested (median 15,536 pg/ml; percentile range 10,631–15,550; normal range 105–1062). No CSF samples were tested for IL-10 or IL-1RA.

### Other soluble markers and combined results

CSF concentration of the chemokine CXCL-13 (C-X-C motif chemokine ligand 13) was tested in a single patient in group I (with cranial nerve and peripheral nerve involvement) and was below the detection limit (< 4 pg/ml). By contrast, CXCL-13 CSF levels were extremely high (38,776 pg/ml) in a patient from group II; however, this patient suffered from primary CNS lymphoma at the time he acquired COVID-19. Serum concentrations of the soluble interleukin-2 receptor (sIL2R), a marker of active cellular immunity, was determined in 4 patients in group I and was elevated in all of them; in total, 13/13 (100%) samples were positive (median 3130 IU/ml; percentile range 1466.6–8894; cut-off 710 IU/ml). Relatively high levels were also found in the aqueous humor in one patient (1068 IU/ml), in whom CSF was not tested. Serum neopterin was tested in two patients in group I and was elevated in both (5.04 and 6.48 ng/ml, respectively; normal < 2.5 ng/ml). Interleukin-17 (IL-17), an important mediator of the mucosal immune response, was not directly assessed in CSF or serum, but IL-17 secretion after whole blood stimulation with *Staphylococcus aureus* enterotoxin B (SEB) for 24 h, a marker of lymphocyte function/immunodeficiency, was assessed in 10 samples from 10 patients in group I. Interestingly, decreased IL-17 secretion was found in 4 samples from 4 patients (normal values in the remainder), none of whom was treated with immunosuppressants or steroids at the time of testing.

In 9 patients, blood levels of 3 cytokines and cytokine receptors (from the following panel: IL-6, IL-8, TNF-alpha, IL-1b, sIL2R, IL-10, IL1RA) were assessed; in 8 of these, increased levels of all three were observed at least once and in 1 patient increased levels of two markers. In another 11 patients, blood levels of 5 markers from the same panel were determined; in 4 of these, levels of all 5 markers were elevated; in 5 samples those of four markers, and in 2 samples those of at least two markers. In a further patient, 6 of the beforementioned cytokine markers were assessed in the blood and all were elevated.

Not only IL-6 blood levels remained detectable over weeks or months but also those of other cytokines. IL-6, IL-8, TNF-alpha, IL-10 and sIL2 were still elevated in 12/13 (92.3%), 4/4, 5/6, 4/4 and 1/1 samples taken > 14 days after onset of the patients' neurological symptoms, respectively, and in 20/21 (95.2%), 6/7, 8/11, 3/3 and 3/3 samples obtained ≥30 days (median 40.5; range 30–205) after onset of the first non-neurological symptoms of COVID-19. CSF IL-6 and IL-8 were also still elevated in the repeat sample with the longest follow-up tested, which was obtained 16 days after neurological and 35 days after non-neurological COVID-19 onset.

### First vs. follow-up LP

The frequency of OCBs, pleocytosis, BCB dysfunction, CSF TP elevation, and CSF l-lactate elevation did not differ significantly between the first LP and follow-up LP (median time interval between punctures was 13 days) in group I (Table 8). All but one of the follow-up samples were obtained from patients in the B/SC subgroup. The proportions of patients with severe, moderate, or mild disease, respectively, among those with repeat LP did not differ significantly from those among the remaining patients (severe: 54% vs. 60%, moderate: 31% vs. 22%, severe or moderate: 85 vs. 82%; mild: 15 vs. 18%).

**Table 8:** CSF findings at the time of the first LP and at follow-up LP in group I

	Units	First LP	Follow-up LPs
Pleocytosis, all	Samples	11/113 (9.7%)	3/15 (20%)
Pleocytosis, B/SC subgroup	Samples	10/92 (10.9%)	2/14 (14.3%)
Pleocytosis, PN/CN/H subgroup	Samples	1/20 (5%)	1/1 (100%)
OCB, all	Samples	2/92 (2.2%)	0/11 (0%)
OCB, B/SC subgroup	Samples	2/75 (2.7%)	0/10 (0%)
OCB, PN/CN/H subgroup	Samples	0/16 (0%)	0/1 (0%)
IgG-IF > 10%, all	Samples	1/104 (1%)	0/11 (0%)
IgG-IF > 10%, B/SC subgroup	Samples	1/83 (1.2%)	0/11 (0%)
IgG-IF > 10%, PN/CN/H subgroup	Samples	0/20 (0%)	Not done
QAlb > Qlim(Alb), all	Samples	51/104 (49%)	7/12 (58.3%)
QAlb > Qlim(Alb), B/SC subgroup	Samples	42/83 (50.6%)	7/12 (58.3%)
QAlb > Qlim(Alb), PN/CN/H subgroup	Samples	8/20 (40%)	Not done
CSF TP elevated, all	Samples	47/104 (45.2%)	7/14 (50%)
CSF TP elevated, B/SC subgroup	Samples	41/87 (47.1%)	7/14 (50%)
CSF TP elevated, PN/CN/H subgroup	Samples	6/16 (37.5%)	Not done
CSF L-lactate elevated, all	Samples	23/96 (24%)	3/13 (23.1%)
CSF L-lactate elevated, B/SC subgroup	Samples	19/80 (23.8%)	3/12 (25%)
CSF L-lactate elevated, PN/CN/H subgroup	Samples	3/15 (20%)	0/1 (0%)
Time since onset of neurological symptoms	Days	5 (0–82)	16 (2–38)

*B/SC* brain/spinal cord, *IgG-IF* intrathecal IgG fraction, *OCB* oligoclonal bands, *PN/CN/H* peripheral nerve/cranial nerve/headache only, *QAlb* CSF/serum albumin quotient, *TP* total protein, *WCC* white cell count

A total of 13 repeat tests for OCB were performed in 10 patients. No change in OCB status or OCB pattern (6 × pattern 4, 2 × pattern 1) over time was noted. Similarly, 8/8 (100%) patients who were tested more than once had a normal IgG CSF/serum ratio both at first LP and at follow-up. An intrathecal ‘IgM to IgG switch’ was observed in 0/8 patients in whom QIgM and QIgG were determined more than once (median period since first LP: 14 days, range 2–33).

### Disease severity

In patients classified as having ‘severe’ or ‘moderate’ neurological disease at the time of LP by the treating physicians, many CSF parameters were more markedly or more frequently altered than in patients classified as having mild disease, including the frequency of OCB pattern 4 (as opposed to OCB pattern 1, i.e., a lack of OCB, which was more common in ‘mild’ disease), median QIgG and QAlb levels, the frequency of BCB dysfunction, median TP levels in those with elevated TP, the frequency of TP values > 100 mg/dl, median l-lactate levels in those with and those without elevated l-lactate, and the frequency of samples with elevated l-lactate (Table 9). As pleocytosis was generally rare and, if present at all, mild in the vast majority of cases, no significant difference in WCC was found between the two groups.

**Table 9:** CSF findings and attack severity in patients with acute disease in group I (i.e., ≤ 14 days since onset of the neurological symptoms)

	Units	Severe/moderate	Mild
WCC, all	Cells/µl	2 (0–651;71)	2 (0–373;15)
WCC, elevated	Samples	10/62 (16.1%)	1/7 (14.3%)
OCB, pattern 1	Samples	26/80 (32.5%)	13/20 (65%)
OCB, pattern 4	Samples	50/80 (62.5%)	4/20 (20%)
Link index	Samples	1/65 (1.5%)	0/14 (0%)
QIgG, all	Ratio	4.3 (1–22.4;66)	2.9 (1.6–7.5;14)
QIgG, elevated	Samples	1/66 (1.5%)	0/14 (0%)
QAlb, all	Ratio	9.3 (3.2–50.8;66)	6.9 (3.6–29.9;15)
QAlb, elevated	Samples	39/66 (59.1%)	7/15 (46.7%)
QAlb, if elevated	Ratio	12.5 (6.8–50.8;39)	9.8 (6.7–29.9;7)
CSF TP, all	Mg/dl	49 (18.2–240.4;63)	42.2 (17.5–175;13)
CSF TP, elevated	Samples	35/63 (55.6%)	6/13 (46.2%)
CSF TP, if elevated	Mg/dl	70.1 (45.3–240.4;35)	54.7 (48.6–175;6)
CSF TP, > 100 mg/dl	Samples	11/90 (12.2%)	1/18 (5.6%)
CSF L-lactate, all	Mmol/l	2.1 (1.3–4;60)	1.7 (1.3–3.5;13)
CSF L-lactate, elevated	Samples	16/60 (26.7%)	4/13 (30.8%)
CSF L-lactate, if elevated	Mmol/l	3.1 (2.2–4;16)	2.8 (2.6–3.5;4)
CSF L-lactate, > 3 mmol/l	Samples	11/85 (12.9%)	2/18 (11.1%)

Results are given as medians (with ranges and sample or patient numbers in brackets) and frequencies (with percentages in brackets), respectively. *OCB* oligoclonal IgG bands, *QAlb* CSF/serum albumin quotient, *QIgG* CSF/serum IgG quotient; *TP* total protein, *WCC* white cell count

### Impact of immunotherapy

Several CSF parameters were either pathologically altered only in samples from patients untreated at the time of LP (N = 89) or more severely pathologically altered in these samples than in samples from patients treated with steroids, immunosuppressants or immunomodulatory drugs at the time of LP (N = 29) in group I (e.g., pleocytosis: 13/89 [14.6%] vs. 1/29 [3.4%]; intrathecal IgG, IgA, or IgM synthesis: 6/70 [8.6%] vs. 0/24 [0%]; higher CSF and serum IgG, IgM, IgA concentrations; higher QAlb values in those with BCB dysfunction; higher TP and l-lactate concentrations in those with elevated values). However, the differences did not reach statistical significance. The steroids used included methylprednisolone, prednisone and dexamethasone (*N* = 26); the immunosuppressive or immunomodulatory treatments used included intravenous immunoglobulins, sulfasalazine, tacrolimus, mycophenolate mofetil, and ciclosporin A and were given either alone (*N* = 3) or in combination with steroids (*N* = 3).

Of note, BCB dysfunction, the most frequent pathological finding in this cohort, as well as related alterations (ACD rate, rate of samples with elevated TP, samples with TP concentrations > 100 mg/dl) were found with equal or even higher frequency in samples from treated patients versus untreated patients. This could partly reflect the fact that the subgroup of treated patients included more patients with severe disease and/or a delayed or insufficient effect of the treatments applied on these parameters. Of note, WCC was rarely elevated in general in this cohort, including in untreated patients, which would in any case render it highly difficult to detect treatment effects on the rate of samples with ACD. To explain the apparent lack of effect on BCB function and related findings, detailed analysis would be required of all individual cases taking account of steroid timing, dosage, and the severity of BCB dysfunction, which was not part of the study protocol.

### ‘Normal’ CSF

A substantial number of CSF samples in group I exhibited no pathological changes. If CSF WCC, OCB, QIgG, Link index, QIgM, QIgA, QAlb, CSF TP, and CSF L-lactate are taken into account, 28 (35%) of 80 samples in group I in which all of these 9 parameters were assessed showed exclusively normal values. Of these 28 samples, 17 were obtained ≤14 d and 7 > 14 days after onset of neurological symptoms (no data in 4). If only a basic panel consisting of CSF WCC, CSF TP, and CSF L-lactate is considered (reflecting clinical practice in some non-tertiary centers and in emergency room settings), 49 (46.2%) of 106 samples in which all of these 3 parameters were determined samples would have been classified as 'normal'. Of those, 42.9% (21/49) would have been false-negatives (with the more extensive panel of 9 CSF parameters serving as gold standard).

### Quotient diagrams (‘reibergrams’)

**Fig. 9:**
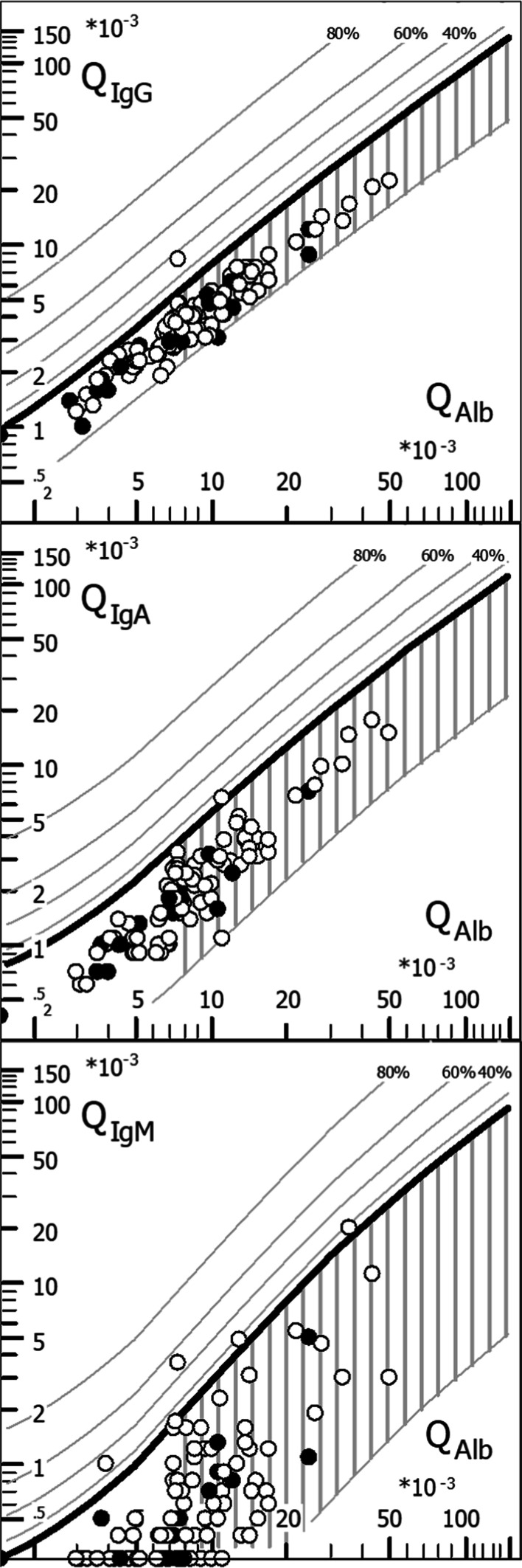
CSF/serum quotient diagrams for IgG, IgM, and IgA (‘reibergrams’). Individual CSF/serum ratios of IgG, IgA, and IgM are plotted against CSF/serum albumin ratios. Values above the upper hyperbolic discrimination line, Q_lim_, indicate intrathecal synthesis of the respective immunoglobulin (Ig) class. Individual intrathecal fractions, Ig_IF_, can be directly read by interpolation from the percentiles above Qlim (median values are given in Tables 3 and 4). Open circles represent samples from the ‘B/SC subgroup’; filled circles represent samples from the ‘PN/CN/H subgroup’. Graphs were created using *CSF Research Tool* v3.0 (CoMed GmbH, Soest, Germany). *B/SC* brain spinal cord, *IgG/A/M* immunoglobulin G/A/M, *PN/CN/H* peripheral nerve/cranial nerve/headache only, *QIgG/A/M* CSF/serum IgG/A/M ratios, *QAlb* CSF/serum albumin ratio

Plots of QIgG, QIgA, and QIgM [21, 23], respectively, against QAlb (as a measure of BCB function), visually indicating absence of intrathecal IgG, IgM, and IgA synthesis in most cases (as evident from the fact that most quotients are below the upper hyperbolic discrimination line, Q_lim_, of the respective immunoglobulin class), are shown for group I in Fig. 9 (see figure legend for details). Note that reibergrams, as a limitation, permit visualizing the frequency of BCB dysfunction only for individual patients or for groups of patients of the same age, but not at population level, since the upper reference limit of QAlb is age-dependent; in the plots shown in Fig. 9, the gray vertical lines indicate the area above the upper limit of QAlb for the median age in group I, which was 65 years.

#### CSF findings in group II

CSF findings in patients with pre- or co-existing CNS disorders did not substantially differ from those typically present in patients with the respective disorders and no COVID-19.

In group II, positive pattern 2 or 3 OCB, which were extremely rare in group I (~ 1%), pointed to the presence of coexisting MS (3 patients; 1 × with complicating John Cunningham [JC] virus-positive progressive multifocal leukoencephalopathy [PML] and a subsequent immune reconstitution inflammatory response [IRIS] after withdrawal of natalizumab) or viral meningoencephalitis (1 × HSV, 1 × VZV, 1 × HSV and CMV in an immunosuppressed patient). A bispecific MRZ reaction was noted in none of the patients in group I but was present in the only MS patient in group II tested for this marker. However, QAlb values were higher than expected (15 × 10^–3^; typically < 12 × 10^–3^ in MS) in one case, probably due to additional JC virus-associated (and possibly SARS-CoV-2-associated) BCB dysfunction.

HSV and VZV meningoencephalitis was associated with pleocytosis in 3/3, higher WCC at first LP (73, 177, 32 cells/µl) than observed in 97% of all patients in group I, CSF-restricted OCB in 2/3 patients, and BCB dysfunction in 3/3 with QAlb values (higher than those in 90% of patients in group II). In an immunosuppressed patient (after kidney transplantation), CSF PCR for CMV and HSV1/2 as well as serum PCR for CMV, HSV1/2 and EBV were all positive, further underlining the need for extensive PCR diagnostics in patients presenting with COVID-19 and neurological symptoms. Neutrophilic (70%) pleocytosis (32 cells/µl) was also noted in this patient, probably because septic brain infarction has occurred as a result of acute bacterial endocarditis rather than reflecting early viral infection. Neutrophils were found in only 3% of patients with available cytology data in group I and should thus be considered a red flag. In a further patient with a history of chronic viral meningitis of unknown cause (previous WCC: 186 [2017], 49 [2018] and 83 [2019] cells/µl), COVID-19 and headache, WCC, OCB, QIgG/IgA/IgM and QAlb were all normal; only CSF TP was elevated, albeit markedly so (295 mg/dl; compared with a median of 41.75 mg/dl in group I [all ≤ 240 mg/dl; 95% ≤ 160]).

In patients with additional SAH, high CSF erythrocyte counts (6050–> 100,000), siderophages, and xanthochrome CSF were noted as well as high TP level (up to 300 mg/dl).

Primary CNS lymphoma, present in one patient, was associated with a higher WCC (21 and 88 cells/µl) than observed in most patients in group I, elevated l-lactate levels and marked BCB dysfunction (QAlb 19 and 17, respectively; compared with a median QAlb of 7.9 in group I). In a patient with non-small cell-lung cancer, meningeal carcinomatosis and cerebral metastases, mild pleocytosis (10 cells/µl), and, elevated CSF l-lactate and TP levels were found.

Finally, in a patient with delayed wake-up reaction after sedation, encephalopathy, and myoclonus, a PET-CT-supported diagnosis of anti-Yo-associated autoimmune encephalitis (with positive antibodies in both CSF and serum) was made. CSF analysis revealed severe BCB damage (QAlb 26.7) and, of note, intrathecal synthesis of IgA and IgM (IgM-IF 51.83%, IgA-IF 35.24%), a feature not seen in any of the patients in group I; WCC could be determined only at follow-up and was then mildly elevated (17 cells/µl). 

SARS-CoV-2 PCR was negative in 8/8 CSF samples tested (from 6 patients) in group II.

## Discussion

A substantial proportion of patients with COVID-19 develop neurological symptoms. However, the pathophysiology of such complications is not fully understood. CSF analysis may aid in addressing this question. Furthermore, CSF analysis may be clinically important by virtue of guiding diagnostic and differential diagnostic decisions.

This study, which is, to the best of our knowledge, the largest and most comprehensive on CSF findings in COVID-19 conducted to date, demonstrates that in the majority of cases neurological involvement in COVID-19 is not associated with signs of intrathecal inflammation, as indicated by a normal WCC and a lack of quantitative (immunoglobulin CSF/serum ratios, reibergrams, antibody indices, Link index, FLC-kappa index) or qualitative (CSF-restricted OCB) evidence of total or SARS-CoV-2-specific IgG, IgA, or IgM synthesis within the CNS. Importantly, the remarkable absence of intrathecal inflammation was not restricted to patients with mild disease or those with peripheral symptoms but was found also in almost all patients with severe disease and in the B/SC subgroup.

The median interval between onset of the non-neurological COVID-19 symptoms or the first positive nasopharyngeal SARS-CoV-2 PCR and LP was just 19 and 12 days, respectively. Moreover, and, most importantly, the neurological symptoms had started only 5 days (median) before LP. It is theoretically possible that intrathecal antibody synthesis began later and was thus not detected. In fact, 10.9% of patients tested had not yet developed SARS-CoV-2 serum antibodies at the time of LP. On the other hand, OCB were missing also in all 21 samples obtained > 14 days after neurological onset and even in all 8 samples taken after more than 30 days (range 31–82), suggesting that the observed lack of intrathecal immunoglobulin synthesis was not simply due to timing. Rather, our findings favor the notion that the neurological symptoms observed in patients with COVID-19 are not caused by direct CNS infection with SARS-CoV-2 in most cases.

This said, it should not go unmentioned that in two patients in group I direct infection of the CNS with SARS-CoV-2 cannot be ruled out. These were the almost only patients with a strongly elevated WCC (247 and 510 cells/µl, compared to a median of 2 cells/µl in group I; neutrophils present in both) and both exhibited at the same time a positive SARS-CoV-2-IgG-AI. Further support in favor of infection of the CNS comes from the finding of a strongly elevated IL-6 CSF/serum ratio in one of them (AI > 17; not tested in the other patient)—no IL-6 ratio (and index) as high was present in any of the SARS-CoV-2-IgG-AI-negative samples tested (which showed a median ratio of just 0.35) –, of a positive TNF-alpha ratio (and high index), and of a positive SARS-CoV-2-IgM AI and SARS-CoV-2-IgA AI in the same patient (not tested in the second patient). In both cases, severe BCB dysfunction was noted. The capability of SARS-CoV-2 to infect neurons has been suggested by a recent study, and neuropathological autopsy findings suggesting CNS infection in some patients with COVID-19 have indeed been published [5, 6].

As a limitation, SARS-CoV-2-CSF-PCR was negative in both cases. PCR was also negative in 82 further samples tested in this study. These findings from a large cohort confirm results from small case series and case reports also describing negative SARS-CoV-2-CSF-PCR results in patients with COVID-19 and neurological symptoms (summarized in [47]) and from a smaller French single-center study that found no unequivocally PCR-positive samples among 23 CSF samples from patients with COVID-19 (two post-mortem CSF samples were borderline positive in that study, but blood contamination was suspected in at least one of these, which was blood-PCR positive) [48].

A negative PCR result does not per se rule out infection of the nervous system. In a recent study, 96/96 Danish CSF samples from patients with encephalitis due to infection with tick-borne encephalitis virus, all of whom met the European Centre for Disease Prevention and Control (ECDC) criteria, were found to be reverse transcriptase (RT)-PCR-negative. In HSVE, a positive CSF PCR result is sometimes obtained only in sequential LP [49–51], probably reflecting both a relatively low viral load in the CSF at onset and the rostro-caudal gradient (lower concentrations in lumbar than in ventricular or cisternal CSF samples) that affects all CSF parameters in patients with CNS disease. In accordance with this notion, SARS-CoV-2-CSF-PCR was initially negative in a patient reported by Huang et al. (2020) but found positive by RT-PCR upon repeat LP [52]. This said, PCR was negative both in the initial and in a follow-up CSF sample in 6 patients of the present cohort and both in samples taken shortly after neurological onset and in samples taken 1–2 weeks later after neurological onset. No ventricular CSF or brain tissue were available for PCR testing in this study.

Similarly, the lack of CSF-restricted total IgG OCB and of quantitative evidence of intrathecal total IgG synthesis in those two patients with a positive SARS-CoV-2-AI and pleocytosis does not formally rule out CNS infection with SARS-CoV-2 either. Total IgG OCB (and also QIgG) are rather markers of chronic inflammation and may be negative at onset. Higher sensitivity of antigen-specific IgG AI than of markers of total IgG synthesis such as OCBs and immunoglobulin ratios has been previously shown [53–62]. For example, it has long been known that OCB are detectable only in a subset of VZV-AI-positive patients with (PCR-proven) varicella zoster meningitis or facial paresis[56]. Similarly, positive virus-specific AI and positive antigen-specific OCB, being part of the so-called polyspecific, intrathecal humoral immune response (‘MRZ reaction’), have been found in patients with negative total IgG OCB and normal QIgG in multiple sclerosis [59–62].

We recommend that future studies on patients with COVID-19 and neurological symptoms should generally consider not only WCC, PCR, and markers of total IgG synthesis such as OCB and QIgG but also the SARS-CoV-2-IgG-AI (and, ideally, the SARS-CoV-2-IgA and -IgM AI), which might have diagnostic and prognostic implications, and, provided sequential LP are performed for clinical purposes, should examine the temporal dynamics of the intrathecal antiviral immune response in more detail. As the median interval between onset of neurological symptoms and AI determination was relatively short in the present cohort (median 5, range 1–49 days), results from follow-up LPs may be of particular interest. Based on the finding of high IL-6 and TNF-alpha CSF/serum ratios and indices in the only SARS-CoV-2-IgG-AI-positive patient tested but not in any other patients, such studies should evaluate also IL-6 and TNF-alpha as supplementary markers of acute intrathecal inflammation in COVID-19. Finally, also potential cross-reactivity between anti-SARS-CoV-2 antibodies and neuronal antigens (as recently discussed [63]) could explain the rare finding of a positive SARS-CoV-2 AI in the absence of a positive SARS-CoV-2 PCR and deserves to be investigated in more detail.

A striking feature in this cohort was the high frequency of BCB dysfunction. Disturbed BCB integrity, indicated by an increased albumin CSF/serum ratio, was present not only at onset but also in samples taken more than 30 days later. The presence of BCB dysfunction sufficiently explains the elevated CSF immunoglobulin and free kappa and lambda light chain concentrations and the high total TP concentrations found in some patients, as indicated by a highly significant correlation of both CSF IgG and CSF TP with QAlb and the lack of QIg elevation.

Most importantly, in most cases BCB dysfunction was not associated with signs of cellular or humoral inflammation. Accordingly, a high rate of ACD was found (around 40% of all samples and around 80% in those with elevated QAlb). It is thus likely that BCB dysfunction was not primarily a result of intrathecal inflammation in our patients. The generally high rate of ACD needs also to be considered as a caveat when diagnosing patients presenting with COVID-19 and signs and symptoms of peripheral nerve disease. While ACD is an important marker of GBS, its predictive value for that disease may be lower in patients with COVID-19.

Little is known about the exact mechanisms leading to impaired BCB function in COVID-19. These may include direct infection (and subsequent apoptosis) of endothelial cells and other cells constituting the blood–brain barrier (BBB) and the anatomic BCB with SARS-CoV-2 as well as nonspecific inflammatory bystander damage to these structures [64–67], SARS-CoV-2-induced peripheral antiendothelial autoimmunity [68] (reported also after SARS-CoV-1 infection [69]), and hypoxia-related damage. In fact, severe endothelial cell damage has been repeatedly described in COVID-19 patients [65, 70, 71] (as well as a capillary-leak syndrome in rare cases [72, 73]).

Given the existing reports on severe peripheral inflammation in COVID-19 and a cytokine storm in the blood of severely affected patients, inflammatory bystander damage of peripheral origin to the BCB seems at least conceivable. Blood leukocytosis is present in many patients with acute COVID-19, and a ‘mirror OCB pattern’ (also termed pattern 4), which may indicate peripheral, polyclonal acute inflammation, was present in 56% of our patients. Moreover, inflammatory cytokines, including markers indicating cellular inflammation, were frequently and persistently elevated in many patients in this cohort at the time of LP, with very high values in some. The spectrum of increased soluble markers of inflammation observed in our patients was broad, including both proinflammatory (such as IL-6, IL-8, IL1-beta, TNF-alpha and IFN-gamma) and anti-inflammatory (IL-10 and IL-1RA) cytokines and cytokine receptors. In some patients extremely high serum IL-6 levels were noted, suggesting severe systemic inflammation, and IL-6 remained persistently positive over time in > 95% of more than 700 samples taken over a median period of around 1 month during COVID-19-related hospitalization. This broad and strong activation of inflammatory pathways renders it likely that other mediators of inflammation implicated in BBB disruption not investigated in this cohort such as ROS (reactive oxygen species), NETs (neutrophil extracellular traps), MMPs (matrix metalloproteinases) and complement may have been elevated as well. In addition, hypoxia- or acidosis-related damage to the BCB in ICU-treated patients [74], who account for a high percentage of those included in this study, cannot be ruled out; in fact, a slightly (albeit not statistically significant) higher rate of BCB dysfunction was found in ICU-treated patients (58 vs. 41.5%). Finally, QAlb elevation and ACD were associated with GBS (*N* = 3) or brain infarction/bleeding (*N* = 7), conditions known to cause BCB dysfunction, in a relatively small subset of patients in this cohort.

An alternative (or additional) explanation for QAlb elevation is a change in CSF flow rate [25], which could, hypothetically, be caused by decreased CSF production or CSF resorption (e.g. due to infection [75–77] or inflammatory bystander damage affecting the function of the choroid plexus, the arachnoid granulations, or the cranial and/or spinal nerve sheaths).

CSF l-lactate levels were elevated in a quarter of our patients, based on age-dependent reference ranges, with a trend towards higher levels in acute samples. Physiologically, CSF l-lactate levels are relatively independent from serum l-lactate levels due to saturated lactate transporters. CSF l-lactate correlated significantly with QAlb, however, and the median l-lactate CSF level was significantly higher and CSF-lactate elevation significantly more common in patients with BCB dysfunction, suggesting a possible contribution of serum l-lactate to CSF level. Regrettably, l-lactate levels were not determined in the serum. However, CSF levels are usually higher than serum levels, rendering it more likely that CSF l-lactate and QAlb independently reflect the extent of CNS damage.

CSF l-lactate levels were also weakly correlated with the CSF WCC. Among CSF white cells, granulocytes are a known source of CSF l-lactate [78–82]. However, the frequency of pleocytosis was low and only few patients had neutrophils in the CSF. Accordingly, the frequency of l-lactate elevation did not differ between samples with and without granulocytes and no significant correlation between CSF granulocyte counts and CSF l-lactate levels was found in the present cohort. CSF l-lactate is thought to be produced also by astrocytes, which form an essential part of the BBB, following glutamate stimulation [83, 84].

Finally, neurons may switch to glycolysis, in particular when their capacity to metabolize anaerobically the lactate of astrocytic origin is exhausted [84]. The elevated l-lactate levels may thus reflect cerebral hypoxia due to COVID-19-induced pneumonia or cerebral ischemia [85, 86]. CSF l-lactate levels were indeed significantly higher in the subgroup of patients requiring ventilation at the time of LP or within 1 week before or after LP. Further studies are needed to better characterize the sources of intrathecal l-lactate in COVID-19.

The relatively high frequency of normal CSF findings means that altered CSF parameters in patients presenting with COVID-19 and neurological symptoms, in particular pleocytosis, intrathecal immunoglobulin synthesis and unusually high QAlb values, should always prompt clinicians to consider the presence of pre- or coexisting conditions, which may require specific treatment and must thus not be overlooked or mistaken for direct effects of COVID-19. In fact, a broad panel of coexisting neurological diseases was present in this cohort, which is hardly astonishing given the currently high prevalence of COVID-19. Importantly, these patients were analyzed separately (group II). The CSF findings in this subgroup were mainly representative of the respective comorbidity.

From a clinical point of view, it is also important to keep in mind that restricting CSF analysis to a basic panel (CSF WCC, TP, and l-lactate), as is often practiced in non-tertiary care or emergency settings, was associated with a relatively high rate of false-negative reports in our cohort. We strongly recommend applying a more complete panel including QAlb and, to rule out differential diagnoses, also QIgG, QIgA and QIgM, whenever possible.

While the absence of signs of intrathecal inflammation also in almost all samples obtained ≥14 days and even ≥30 days since neurological onset (including 89% and 81% in the B/SC subgroup) suggests that timing issues were not a general confounder, it cannot be fully ruled out that the humoral immune response had simply not yet developed at the time of LP at least in some cases. In HSVE, CSF is normal in 10–20% of cases during the early phase of disease, with a delay of around 1 week in the onset of the antibody response [35, 87, 88]. Moreover, a normal cell count or normal immunoglobulin ratios do not exclude indirect effects on the CNS due to systemic SARS-CoV-2 infection (e.g. induced by hypoxia, thromboembolic events triggered by COVID-19, or cytokines entering the CNS from the periphery, which are known to mediate not only inflammatory but also non-inflammatory effects in the nervous system). Therefore, it is important to underline that the absence of CSF signs of intrathecal inflammation should not lead one to prematurely dismiss patient-reported neurological symptoms as not being related to COVID-19. Of note, a recent study of autopsy specimens from patients with COVID-19 detected SARS-CoV-2 in cortical neurons as well as pathological features suggestive of infection, which were associated with only minimal immune cell infiltrates [6].

A lack of inflammatory pleocytosis and intrathecal total IgG synthesis does also per se not exclude CNS inflammation of autoimmune etiology. For example, in myelin oligodendrocyte glycoprotein-IgG-associated encephalomyelitis (MOG-EM) [89], a normal CSF has been observed in ~ 10% of all samples, both in children and adults, strongly depending on lesion sites (more commonly normal in optic neuritis than in brain disease, and least frequently in spinal cord disease) and OCB are missing in around 90% in this condition [13, 14]. Similarly, a lack of intrathecal IgG synthesis has been observed in the vast majority of patients with AQP4-IgG-positive NMOSD [15, 90, 91], in LGI1-IgG-associated encephalitis [92, 93], and in IgLON5-IgG-positive encephalomyelitis [92]), in a subset of patients with other CNS disorders of supposed autoimmune etiology such as Balo’s concentric sclerosis [17], Schilder’s disease [18], histopathologically defined “pattern II” and “pattern III” MS [16].

It is of interest in this context, that antibodies to neural antigens have been reported in some patients with COVID-19 [94, 95], rendering it theoretically possible that virus-induced autoimmunity plays a role in a subset of patients with COVID-19 and neurological symptoms. However, independent confirmation of specific autoantibodies from larger and independent cohorts is widely lacking, as is information about target antigens in patients with supposed post-COVID-19 autoimmunity. In the present cohort, 1413 individual tests for established anti-neuronal antibodies were performed in the CSF and serum but were almost all negative. Moreover, 41 samples were in addition tested on nervous tissue sections and did not reveal evidence of unknown autoantibodies. Timing may be critical, however, and follow-up studies, especially in those with long-term sequelae (‘long COVID-19’) are certainly warranted. This is the largest number of tests for antineural antibodies reportedso far in studies on COVID-19.

The fact that BCB dysfunction and elevated levels of inflammatory cytokine levels (especially IL-6) persisted over weeks and months, respectively, is of interest in light of the many reports of patients with ‘non-specific’ long-term sequelae of COVID-19 such as malaise, fatigue, or cognitive impairment. Studies addressing the relevance of cytokines and BCB disruption in ‘long COVID’ are currently underway.

Of note, HSVE and VZV meningoencephalitis, respectively, were present in two patients. Whether infection with SARS-CoV-2 may promote reactivation of latent herpes virus infection remains to be investigated. While a number of reports on COVID-19-associated VZV (and, in one case, HSV-1 [96]) reactivation exist [97–99], including a case of suspected VZV encephalitis [100], coincidence cannot be ruled out given the high current prevalence of COVID-19. A further patient in the present series suffered from JC-virus induced PML. These findings underline the importance of considering the presence of additional, coexisting viral disease in all patients diagnosed with COVID-19. The detection of SARS-CoV-2 infection should not prompt one to dispense additional microbiological laboratory diagnostics. Rather, the diagnostic work-up in patients with proven SARS-CoV-2 infection and neurological symptoms should always be supplemented by PCR and AI analyses for coexisting viral infections.

### Strengths and limitations

We consider the high number of samples included and the availability of follow-up samples as particular strengths of this study, together with the high number of parameters assessed. A further strength is the stratification according to the presence of pre- or coexisting disorders, which helped to identify CSF alterations attributable to conditions other than SARS-CoV-2 infection.

The retrospective and multicenter design of this study is a potential limitation. However, LP is an invasive and potentially harmful procedure that widely precludes prospective studies. The multicenter design can also be viewed as a strength, since it helped to reduce potential center-specific selection bias. Moreover, CSF diagnosis is highly standardized in central European university CSF laboratories, with centers participating in regular round-robin tests required for obtaining quality certification. Finally, given the relatively low prevalence of neurological involvement severe enough to justify LP in COVID-19, a multicenter approach was necessary for inclusion of a sufficiently large number of patients in a short time, which was important given the current pandemic situation.

It is a limitation that some parameters, such as anti-neural autoantibodies, interleukins, interleukin receptors, chemokines, and kappa and lambda light chains, are not routinely assessed at some centers and, accordingly, data were available only for a subset of patients. It is therefore unknown whether the results reported here are representative for the total cohort. While we provide a large dataset for some of these parameters (1509 individual cytokine measurements; 939 individual autoantibody test results), more studies investigating these parameters are certainly warranted, for which our results may provide a rationale. Similarly, no predefined criteria for performing LP were used. Instead, the decision for LP was made in a clinical ‘real-life’ context based on the patient’s clinical and paraclinical presentation. While this could theoretically have introduced referral bias (e.g., if thresholds for performing LP were higher at some centers), this is unlikely given that all LPs were performed in the setting of academic tertiary care hospitals with similar standards of care. CSF analysis is part of the general routine workup of patients with neurological symptoms of unknown cause in Germany, Austria, Switzerland, and Italy.

A trend towards more frequent and more severe CSF alterations in patients classified as having ‘severe’ or ‘moderate’ neurological deficits at the time of LP by the treating centers than in those with ‘mild’ deficits was noted. However, no standardized criteria for disease severity could be applied due to the retrospective nature of this study and the plethora of manifestations present. Further studies are needed to better define the relationship of disease severity and CSF findings in patients with COVID-19 and neurological involvement.

Finally, as this study was performed at European centers, most patients were of ‘Caucasian’ origin. It is unknown if our results are valid also for other ethnicities.

## Conclusions

In summary, our study demonstrates (i.) that BCB dysfunction, as indicated by elevated QAlb, is the most common pathological CSF feature in patients with COVID-19 and neurological symptoms and may remain present for several weeks; (ii.) that BCB dysfunction in COVID-19 is not typically caused by intrathecal inflammation, as indicated by a lack of pleocytosis (and, correspondingly, a high rate of ACD) and intrathecal IgG synthesis in most patients with elevated QAlb, favoring a peripheral origin (e.g., due to systemic hypoxia, ischemia, elevated serum levels of cytokines and other mediators of inflammation, endothelial cell/choroid plexus infection with SARS-CoV-2, possibly antiendothelial autoimmunity); (iii.) that classical signs of intrathecal CSF inflammation (pleocytosis, CSF-restricted OCB, positive QIgG, QIgA or QIgM) are generally rare in patients with COVID-19 and neurological symptoms, both in samples obtained in the acute phase and in samples obtained later and irrespective of clinical disease severity; (iv.) that the SARS-CoV-2-IgG antibody index, which was elevated in 2 of the 3 only patients with marked pleocytosis (and in 2/20 patients tested), may be a marker of potential interest (alongside the SARS-CoV-2-IgM and -IgA AI) that may indicate either rare SARS-CoV-2 infection of the CNS in COVID-19 or cross reactivity of SARS-CoV-2-IgG with CNS antigens and which may be more sensitive than markers of total IgG synthesis, although more studies on the frequency, diagnostic and prognostic implications, and temporal dynamics of SARS-CoV-2 AI elevation in COVID-19 are needed; (v.) that proinflammatory cytokine levels are frequently elevated in the CSF in patients with COVID-19 and that this often reflects BCB dysfunction rather than intrathecal cytokine synthesis, as indicated by CSF/serum cytokine ratios and indices; (vi.) that the IL-6 and TNF-alpha CSF/serum ratios and indices may be worthwhile being investigated in future studies on CSF findings in COVID-19 as potential markers of CNS infection with SARS-CoV-2, given that the only patient positive also exhibited increased SARS-CoV-2-IgG, -IgM and -IgA AI as well as marked CSF pleocytosis; (vii.) that elevated cytokine levels, like BCB dysfunction, may remain detectable over weeks and months, suggesting a potential role in ‘long COVID’ (or ‘post-COVID’), all the more since some of these cytokines are known to exert also non-inflammation-related undesirable effects in the CNS; (viii.) that anti-neuronal and anti-glial autoantibodies as detectable by routine methodology are rare in patients with COVID-19 and neurological symptoms (although more studies in that regard are required, which should also include patients with ‘long COVID’); (ix.) that SARS-CoV-2-PCR is typically negative in CSF samples from patients with COVID-19 and neurological symptoms (but should not be dismissed since it might prove useful in individual patients) and should always be accompanied by PCR testing for other viral diseases of the CNS, which can coexist with COVID-19, as was the case in several patients of this cohort; (x.) that ‘normal’ CSF findings are not rare (but should not lead to pre-mature dismissal of the presence of COVID-19-related CNS damage, for false-negative results may occur in emergency or non-tertiary care settings when only a basic panel of parameters is assessed and indirect sequelae of COVID-19, e.g., due to ischemia or systemic hypoxia, might not result in marked CSF alterations); and, last but not least, (xi.) the importance of considering differential diagnoses in all patients presenting with neurological symptoms and a positive SARS-CoV-2 swab. Broad CSF analysis, including extensive screening for viruses by PCR as well as for pathological AIs and autoantibodies, is essential to avoid misinterpretation of treatable coexisting neurological disorders as complications of COVID-19. Given the relative rarity of CSF pathology observed in group I, we recommend that markedly altered CSF findings, in particular an increased WCC, should not only prompt SARS-CoV-2-IgG AI and SARS-CoV-2 PCR testing to detect cases of possible CNS infection with that virus but also broad differential diagnostic endeavors to identify or rule out co-existing CNS disorders in patients with COVID-19 and neurological symptoms.

## Supplementary Information

**Additional file 1. Table S1. **Patient and subgroup characteristics.

## Data Availability

The datasets generated and/or analyzed during the current study are not publicly available but can be obtained from the corresponding author upon reasonable request.

## Abbreviations

AMPA1/2-R: α-Amino-3-hydroxy-5-methyl-4-isoxazolepropionic acid receptor
AQP4: Aquaporin-4
AQP4-IgG: AQP4 immunoglobulin G
BB: *Borrelia burgdorferi*
BCB: Blood-CSF barrier
CMV: Cytomegalovirus
CASPR2: Contactin-associated protein-like 2
CSF: Cerebrospinal fluid
DNER: Delta and Notch-like epidermal growth factor-related receptor
DPPX: Dipeptidyl-peptidase-like protein-6
EBV: Epstein Barr virus
IF: Intrathecal fraction
GABA-B1/2-R: G-protein coupled receptors B1/2 for gamma-aminobutyric acid
GAD65: Glutamic acid decarboxylase 65
GluR5: Metabotropic glutamate receptor 5
HHV6: Human herpes virus 6
HSV: Herpes simplex virus
HSVE: HSV encephalitis
IgG/M/A: Immunoglobulin G/M/A
IgLON-5: IgLON family member 5
IS: Intrathecal synthesis
LG1: Leucine-rich, glioma inactivated protein 1
LP: Lumbar puncture
MOG: Myelin oligodendrocyte glycoprotein
EM: Encephalomyelitis
nAChR: Nicotinergic (i.e. ganglionic) acetylcholine receptors
NMDA-R: N-methyl-D-aspartate receptor
NMO: Neuromyelitis optica
NMOSD: NMO spectrum disorders
OCB: Oligoclonal bands
ON: Optic neuritis
QAlb: Albumin CSF/serum ratio
QIgG: IgG CSF/serum ratio
QIgM: IgM CSF/serum ratio
QIgA: IgA CSF/serum ratio
SOX1: SRY-box transcription factor 1
VZV: Varicella zoster virus
Zic4: Zic family member 4
