# Supplementary material for: Cerebrospinal fluid findings in COVID-19: a multicenter study of 150 lumbar punctures in 127 patients
Source: J Neuroinflammation. 2022 Jan 20;19:19. doi: 10.1186/s12974-021-02339-0 (PMC8771621; doi:10.1186/s12974-021-02339-0)
Supplement: Supplementary file 1 — Additional file 1. Table S1. Patient and subgroup characteristics. [file 12974_2021_2339_MOESM1_ESM.docx]

Additional file 1: Table S1. Patient and subgroup characteristics.

| GROUP I (116 patients, 131 samples) |  |  |
| --- | --- | --- |
| Sex |  |  |
| Male | *Patients* | 90 |
| Female | *Patients* | 26 |
| Female/male | *Ratio* | 1:1.41 |
| Age at the time of LP |  |  |
| Median | *Years* | 65 |
| Range | *Years* | 19-85 |
| Ethnic origin |  |  |
| Caucasian | *%* | 88 |
| African | *%* | 5 |
| Turkish/Middle-Eastern | *%* | 4 |
| Asian | *%* | 3 |
| Manifestations at the time of LP (more than one per patient possible): |  |  |
| Encephalopathy/disturbed consciousness | *Samples* | 63 |
| Seizures/epilepsy-like EEG changes | *Samples* | 27 |
| Cerebral ischemia/bleeding | *Samples* | 14 |
| Myelitis | *Samples* | 3 |
| Other CNS manifestations | *Samples* | 25 |
| PNP (incl. GBS) | *Samples* | 22 |
| Cranial nerves | *Samples* | 10 |
| Headache | *Samples* | 13 |
| Clinical subgroups (based on manifestations at the time of LP) |  |  |
| 'B/SC' subgroup | *Samples* | 108 |
| <=14 days between neurological onset and LP | *%* | 75 |
| 'PN/CN/H only' subgroup | *Samples* | 22 |
| <=14 days between neurological onset and LP | *%* | 89 |
| Immunotherapy-related subgroups |  |  |
| Untreated at the time of LP | *Samples* | 89 |
| Treated at the time of LP | *Samples* | 29 |
| Severity of the neurological symptoms, as per judgment of the |  |  |
| treating centers, at the time of LP |  |  |
| 'Mild' | *%* | 17.4 |
| 'Non-mild' (i.e., 'moderate' or 'severe') | *%* | 82.6 |
|  |  |  |
| GROUP II (11 patients, 19 samples) |  |  |
| Sex |  |  |
| Male | *Patients* | 5 |
| Female | *Patients* | 6 |
| Female/male | *Ratio* | 1:0.8 |
| Age at the time of LP |  |  |
| Median | *Years* | 54 |
| Range | *Years* | 29-76 |
| Ethnic origin |  |  |
| Caucasian | *%* | 82 |
| African | *%* | 0 |
| Turkish/Middle-Eastern | *%* | 9 |
| Asian | *%* | 9 |

Abbreviations: *B/SC* brain/spinal cord, *PN/CN/H* peripheral nerve/cranial nerve/headache only, *CNS* central nervous system, *EEG* electroencephalography, *GBS* Guillain‒Barre syndrome, *LP* lumbar puncture, *PNP* peripheral neuropathy.
